# Gasdermin D‐Mediated Pyroptosis Exerts Two Opposite Effects of Resisting Enzymatic Digestion and Expanding Inflammatory Response in Acute Pancreatitis

**DOI:** 10.1002/advs.202502412

**Published:** 2025-05-29

**Authors:** Chaoxu Liu, Ning Liang, Chen Wu, Zhaoyan Qiu, Qian Huang, Xiaolong Wei, Shurong Zhang, Shuanghong Lei, Tao Yang, Gang Wang, Qian Wang

**Affiliations:** ^1^ Department of Colorectal Surgery The First Affiliated Hospital of Zhejiang University Hangzhou 310006 China; ^2^ Department of Gastroenterology 920th Hospital of the Joint Logistics Support Force PLA Yunnan Kunming 650032 China; ^3^ Department of General Surgery Huashan Hospital, Fudan University Shanghai 201907 China; ^4^ Department of General Surgery the First Medical Centre Chinese PLA General Hospital Beijing 100853 China; ^5^ Department of Gynaecology and Obstetrics The 75th Group Army Hospital Dali 671000 China; ^6^ Anorectal Department The First People's Hospital of Longnan Longnan 742500 China; ^7^ Department of Interventional Radiology Tangdu Hospital Fourth Military Medical University Xi'an City Shaanxi Province 710038 China; ^8^ Department of General Surgery Tangdu Hospital Fourth Military Medical University Xi'an Shaanxi 710038 China

**Keywords:** acute pancreatitis, gasdermin D, mucin 1, pyroptosis, trypsin

## Abstract

Gasdermin D (GSDMD)‐induced pyroptosis is associated with inflammatory disease. However, the role of GSDMD in acute pancreatitis (AP) is not yet fully elucidated. This study reveals that GSDMD serves two distinct functions in pancreatic acinar cells and macrophages. In acinar cells, GSDMD inhibits the synthesis of pancreatic enzyme by downregulating the expression of Prss1, Pnlip, and Amy1 via the inhibition of the protein kinase B (AKT)/mammalian target of rapamycin (mTOR)/ribosomal protein S6 (RPS6)/eukaryotic translation initiation factor 4E‐binding protein 1 (4EBP1) pathway. Moreover, GSDMD induces pancreatic acinar cells to express mucin 1 (MUC1) by activating the nuclear factor kappa‐B (NF‐κB) pathway, which forms a barrier that prevents digestive enzyme‐mediated digestion. However, GSDMD promotes the secretion of inflammatory cytokines by macrophages during AP. In addition, GSDMD increases the infiltration of macrophages and neutrophils in AP and increases the proportion of Th1 and Th17 lymphocyte subsets in peripheral blood. However, in general, the harmful effect of GSDMD in AP outweighs its beneficial effect, and GSDMD knockout can effectively alleviate AP. These findings indicate that GSDMD may be a potential target for the treatment of AP; however, its dual effects need to be comprehensively considered.

## Graphical abstract

Gasdermin D (GSDMD) does not only reduce pancreatic enzyme synthesis but also induces pancreatic acinar cells to express mucin 1 (MUC1), which forms a barrier to prevent digestive enzyme‐mediated digestion. However, GSDMD can promote the secretion of inflammatory cytokines by macrophages and aggravate pancreatic histological injury by expanding inflammatory response, which demonstrates two contrasting roles of GSDMD in acute pancreatitis.

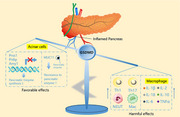

## 1 Introduction

Acute pancreatitis (AP) is a common digestive disease in clinical practice.^[^ ^1^ ^]^ Most patients have mild pancreatitis, with a self‐limiting course and good prognosis. However, ≈20% of the patients may progress to severe pancreatitis, which can be accompanied by systemic inflammatory response syndrome and multiple organ dysfunction syndrome, with a high mortality rate and poor prognosis.^[^ ^1^ ^]^ The global incidence of AP is ≈34 per 100 000 people per year and is on the rise worldwide.^[^ ^2^ ^]^ Although progress has been made in understanding the pathogenesis of AP in the past few decades, there are still no effective drug treatments for AP. At present, the treatment plan for AP is limited to symptomatic treatments such as fasting, spasmolysis, pain relief, and gastrointestinal decompression.^[^ ^3^ ^]^ Therefore, exploring the pathogenesis and treatment of AP is of great importance.

However, its pathogenesis remains unclear. The most widely accepted mechanism is abnormal trypsinogen activation.^[^ ^4^ ^]^ Damaged or dead pancreatic acinar cells release numerous damage‐associated molecular patterns (DAMPs), including adenosine triphosphate, heat shock protein 70, histones, and double‐stranded DNA (dsDNA). Pattern recognition receptors (PRRs), such as Toll‐like receptors (TLRs) and Nod‐like receptors (NLRs), recognize DAMPs released by injured cells, activate the PRR signaling pathway, and cause immune cells, such as neutrophils, macrophages, and T lymphocytes, to migrate to the injured site, triggering a series of inflammatory cascade reactions.^[^ ^5^ ^]^

Presently, gasdermin D (GSDMD) is the most widely studied member of the gasdermin family. There are two pathways for GSDMD activation: the classical inflammasome pathway mediated by caspase‐1 and the nonclassical inflammasome pathway mediated by caspase‐4, ‐5 (human), and ‐11 (mouse).^[^ ^6^ ^]^ In the classical inflammasome pathway, activated caspase‐1 cleaves the variable junction region of GSDMD to form a free N‐terminus, which oligomerizes and forms pores in the cell membrane, leading to pyroptosis.^[^ ^7^ ^]^ In the nonclassical inflammasome pathway, lipopolysaccharides (LPS) from Gram‐negative bacteria can directly activate the caspase recruitment domain (CARD) domain of caspase‐11/4/5 in cells. Activated caspase‐11 cleaves GSDMD to form its N‐terminal fragments, leading to pyroptosis.^[^ ^8^ ^]^ Studies have shown that GSDMD plays a key role in the development of several inflammatory diseases. For example, GSDMD is upregulated in the liver tissues of patients with nonalcoholic fatty liver disease. Besides, GSDMD knockout (GSDMD‐KO) significantly alleviates the progression of steatohepatitis in mice.^[^ ^9^ ^]^ Additionally, GSDMD is involved in the progression of encephalomyelitis^[^ ^10^ ^]^ and colitis.^[^ ^11^ ^]^

Recent studies have shown that GSDMD plays a crucial role in AP development. Specifically, Wu et al. demonstrated that GSDMD knockout improved severe AP (SAP)‐induced pancreatic and related lung injuries.^[^ ^12^ ^]^ Gao et al. found that GSDMD activation‐mediated pyroptosis in acinar cells led to pancreatic necrosis and systemic inflammation in AP.^[^ ^13^ ^]^ In addition, Ma et al. reported that interleukin‐37 prevents acinar cell pyroptosis in AP.^[^ ^14^ ^]^ Moreover, GSDMD is closely associated with the formation of neutrophil extracellular traps in SAP.^[^ ^15^ ^]^ In addition, the NLRP3/caspase‐1/GSDMD pyroptotic pathway is involved in the disruption of the intestinal mucosal barrier in SAP rats, whereas free total rhubarb anthraquinones protects the intestinal mucosal barrier by inhibiting this pathway.^[^ ^16^ ^]^ Presently, various small‐molecule compounds, including necrosulfonamide^[^ ^17^ ^]^ and disulfiram,^[^ ^18^ ^]^ have been found to inhibit GSDMD activity and prevent pyroptosis. In the future, more drugs targeting GSDMD may be discovered, and it will be possible to treat related diseases using GSDMD intervention.

In the present study, we aimed to systematically investigate the significance of GSDMD in humans and experimentally induced AP. We found that GSDMD plays contrasting roles in AP. Regarding the positive role, GSDMD inhibits the synthesis of pancreatic enzyme and develops resistance to digestive enzymes by inducing pancreatic acinar cells to express mucin 1 (MUC1). Regarding the negative role, GSDMD promotes the secretion of inflammatory cytokines by macrophages in AP. In the present study, GSDMD increased the infiltration of macrophages and neutrophils in AP and increased the proportion of Th1 and Th17 lymphocyte subsets in peripheral blood. Therefore, GSDMD may be a potential target for treating AP. However, its two contrasting roles need to be comprehensively considered. Notably, we explored the clinical impact of GSDMD in patients with AP and demonstrated that GSDMD‐mediated pyroptosis is closely related to disease severity, providing new insights into the treatment of AP in humans.

## 2 Results

### 2.1 GSDMD‐Mediated Pyroptosis Is Activated in Human AP and Closely Related to Disease Severity

**Figure 1:**
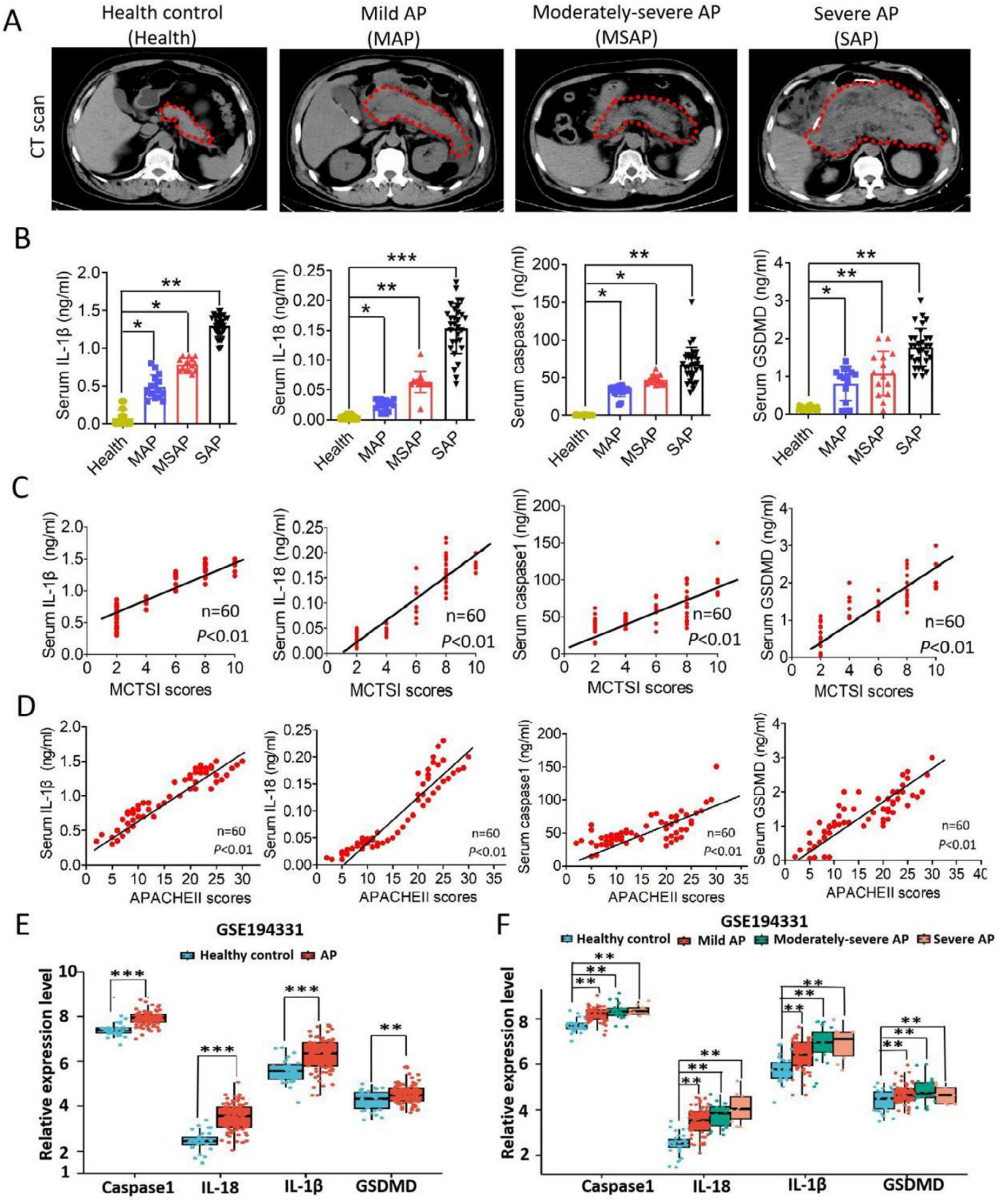
GSDMD‐mediated pyroptosis is activated in human acute pancreatitis and closely related to disease severity. A) Representative computed tomography images of healthy controls, patients with mild acute pancreatitis patients (MAP), patients with moderately severe acute pancreatitis (MSAP), and patients with severe acute pancreatitis (SAP). The pancreatic region was delineated with dashed lines. B) Expression levels of serum IL‐1β, IL‐18, caspase‐1, and GSDMD in healthy controls (*n* = 30) and MAP (*n* = 15), MSAP (*n* = 15), and SAP (*n* = 30) patients. C) Correlation analysis of serum IL‐1β, IL‐18, caspase‐1, and GSDMD levels with MCTSI scores in patients with MAP (*n* = 15), MSAP (*n* = 15), and SAP (*n* = 30). D) Correlation analysis of serum IL‐1β, IL‐18, caspase‐1, and GSDMD levels with APACHE II scores in patients with MAP (*n* = 15), MSAP (*n* = 15), and SAP (*n* = 30). E,F) Transcriptomic profiles of blood samples from 32 healthy controls and 87 AP samples retrieved from GSE194331 confirmed that GSDMD‐mediated pyroptosis is activated in human AP and closely correlated with disease severity. Abbreviation: MCTSI, modified computed tomography severity index; APACHE II, Acute Physiology and Chronic Health Evaluation; AP, acute pancreatitis; GSDMD, gasdermin D. E) *p*‐values were determined by two‐tailed unpaired Student's *t*‐test; B,F) one‐way Analysis Of Variance (ANOVA) with Dunnett's multiple comparisons; and C,D) Pearson's correlation analysis. ***p < 0.001, **p < 0.01, and *p < 0.1.

To investigate the involvement of GSDMD in AP, we first evaluated the expression levels of GSDMD and its downstream activating molecules interleukin‐1beta (IL‐1β), interleukin‐18 (IL‐18), and caspase‐1 in serum samples from 15 patients with mild AP (MAP), 15 patients with moderately severe AP (MSAP), 30 patients with SAP, and 30 control participants. Typical computed tomography (CT) images of each patient are shown in **Figure** **1**A. Compared with the normal control, the expression levels of serum IL‐1β, IL‐18, caspase‐1, and GSDMD were significantly increased in the MAP, MSAP, and SAP groups (Figure 1B). The modified CT severity index (MCTSI) and Acute Physiology and Chronic Health Evaluation (APACHE II) are two widely used tools to assess AP severity. We found that serum levels of IL‐1β, IL‐18, caspase‐1, and GSDMD in AP patients were significantly positively correlated with both MCTSI and APACHE II scores (Figure 1C,D), suggesting that GSDMD‐mediated pyroptosis is closely related to the severity of AP. In addition, the transcriptomic profiles of blood samples from patients with AP retrieved from GSE194331 confirmed that GSDMD‐mediated pyroptosis was activated in human AP and strongly correlated with disease severity (Figure 1E,F).

### 2.2 GSDMD‐Mediated Pyroptosis Is Activated in Mice AP

**Figure 2:**
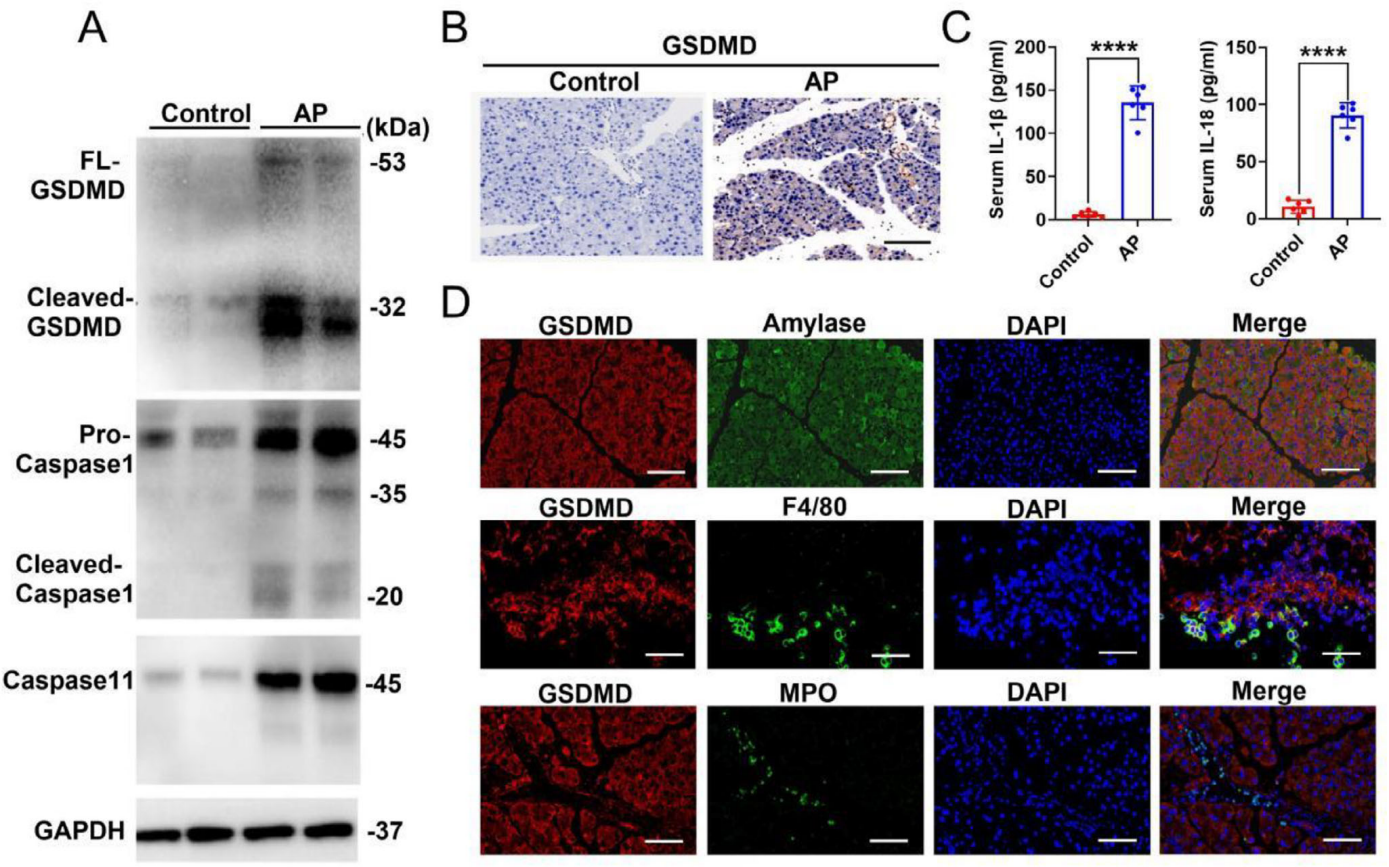
GSDMD‐mediated pyroptosis is activated in mice acute pancreatitis. A) Immunoblotting analysis of the indicated proteins in mouse pancreatic tissues of the control and AP groups. B) Representative IHC images of GSDMD expression in pancreatic tissues of the control (*n* = 6) and AP groups (*n* = 6). Scale bars: 50 µm. C) Expression levels of serum IL‐1β and IL‐18 in the AP group (*n* = 6) compared with those in the control group (*n* = 6). D) Representative immunofluorescence images of GSDMD expression in pancreatic acinar cells, macrophages, and neutrophils. Scale bars: 20 µm. Abbreviation: control, physiological saline control group; AP, acute pancreatitis group; IHC, immunohistochemistry; GSDMD, gasdermin D. C) *p*‐values were determined by two‐tailed unpaired Student's *t*‐test. ****p < 0.0001.

Using western blotting, we compared the expression of GSDMD, caspase‐1, and caspase‐11 in mouse pancreatic tissues of the AP and control groups. Activated caspase‐1 and caspase‐11 can both cleave GSDMD to form N‐terminal fragments, leading to pyroptosis. Our results showed that the activation of the caspase‐1 signaling pathway in the pancreatic tissue of the AP model is more pronounced than caspase‐11, which shows almost no cleavage products. This indicated that upstream caspase‐1 pathways inducing pyroptosis were mainly activated after the occurrence of AP (**Figure** **2**A). Furthermore, immunohistochemical staining confirmed that the proportion of GSDMD‐positive cells in the AP group was significantly higher than that in the control group (Figure 2B). In addition, GSDMD‐mediated pyroptosis markers IL‐1β and IL‐18 were elevated in the serum of the AP group compared to the control group (Figure 2C). In AP mouse models, pancreatic acinar cells showed a high density of anti‐GSDMD immunofluorescence, followed by macrophages and neutrophils (Figure 2D). In addition, immunofluorescence staining showed that caspase‐1 and GSDMD co‐localized in the pancreatic tissue of the AP group, suggesting that GSDMD‐mediated pyroptosis was activated in mouse AP (Figure S1, Supporting Information).

### 2.3 GSDMD Plays Two Contrasting Roles in AP, Increasing Serum Inflammatory Cytokine Levels but Reducing Pancreatic Enzyme Synthesis

**Figure 3:**
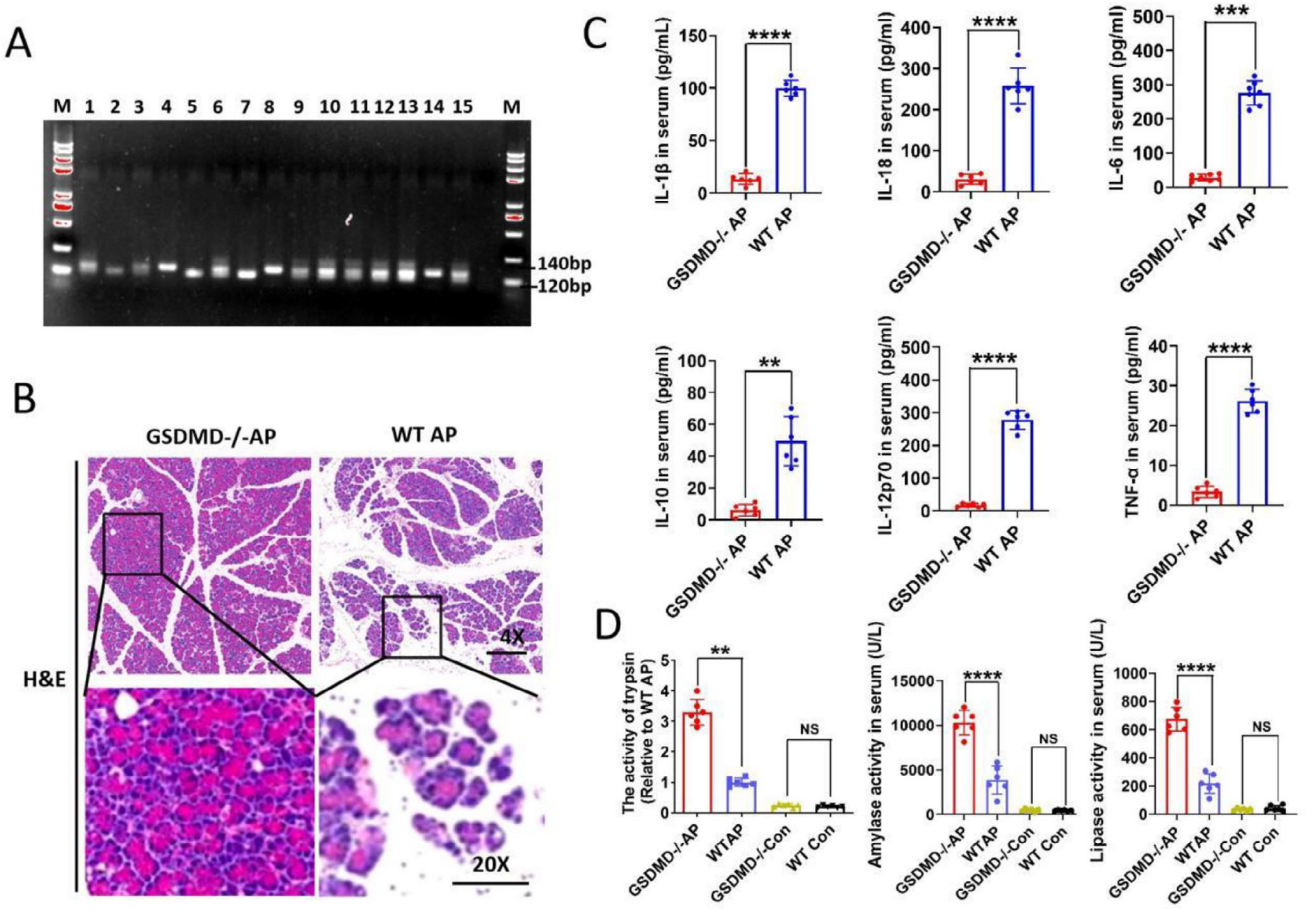
GSDMD plays two contrasting roles in acute pancreatitis, increasing serum inflammatory cytokine levels but reducing pancreatic enzyme synthesis. A) Transgenic mice were identified via polymerase chain reaction (PCR) screening. GSDMD^−/−^: 1 band at 102 bp (#2,5,7); heterozygous: 2 bands of 120 bp and 140 bp (#1,3,6,9,10,11,12,13,15); and the wild‐type: 1 band at 140 bp (#4,8,14). B) Hematoxylin and eosin (H&E) staining of pancreatic tissue sections in the GSDMD^−/−^ AP and WT AP groups. C) Levels of serum inflammatory cytokines in the GSDMD^−/−^ AP (*n* = 6) and WT AP groups (*n* = 6). D) Changes in serum trypsin, amylase, and lipase activities in the GSDMD^−/−^ AP (*n* = 6) and GSDMD^−/−^ Con groups (*n* = 6) and the WT AP (*n* = 6) and WT Con groups (*n* = 6). Abbreviation: NS, not significant; WT, wild type; AP, acute pancreatitis; Con, control; GSDMD, gasdermin D. C) *p*‐values were determined by two‐tailed unpaired Student's *t*‐test; D) one‐way ANOVA with Dunnett's multiple comparisons. ****p < 0.0001, ***p < 0.001, and **p < 0.01.

We generated GSDMD‐knockout mice (GSDMD^−/−^) using the CRISPR/Cas9 method (**Figure** **3**A; Figure S2, Supporting Information). GSDMD knockout did not alter the pathological morphology or inflammatory markers in the pancreas of mice (Figure S3, Supporting Information). To investigate the involvement of GSDMD in AP progression, GSDMD^−/−^ mice and wild‐type (WT) littermates were subjected to intraperitoneal caerulein injection to establish an AP model. Afterward, the pancreas of the WT AP group showed more severe edema and inflammatory infiltration than that of the GSDMD^−/−^ AP group (Figure 3B; Figure S4, Supporting Information). Consistently, the levels of serum inflammatory cytokines such as IL‐1β, IL‐18, IL‐6, IL‐10, IL‐12p70, and tumor necrosis factor‐alpha (TNF‐α) in GSDMD^−/−^ AP group were lower than those in the WT AP group (Figure 3C), suggesting that GSDMD may play a role in promoting inflammation in AP. In contrast, the levels of serum trypsin, amylase, and lipase in the GSDMD^−/−^ AP group were significantly higher than those in the WT AP group, suggesting that GSDMD decreased pancreatic enzyme synthesis in AP (Figure 3D). Taken together, these results suggested that GSDMD plays two contrasting roles in AP. In addition, we utilized adeno‐associated viral vector (AAV)‐based conditional knockout methods to investigate the differential roles of GSDMD in various cell types in vivo. We specifically transfected pancreatic acinar cells with GSDMD‐knockout viral vector through retrograde pancreatic intraductal delivery to elucidate the role of GSDMD in acinar cells. We administered either the hemagglutinin (HA)‐tagged acinar cell‐specific adeno‐associated viral vector (AAV9‐elastase I‐GSDMD‐sgRNA) or a control vector (AAV9‐elastase I‐GSDMD‐sgControl) to the AP mice (Figure S5A, Supporting Information). Importantly, acinar cells expressing the HA‐tag did not display GSDMD staining, while macrophages showed GSDMD staining, confirming a successfully specific knockout of GSDMD in acinar cells (Figure S5B, Supporting Information). Compared to the control group, mice receiving AAV9‐elastase I‐GSDMD‐sgRNA demonstrated an increased synthesis of pancreatic enzymes (Figure S5C, Supporting Information). However, there was no significant difference in serum inflammatory cytokine levels between the two groups (Figure S5D, Supporting Information). Subsequently, macrophage‐specific transfection with GSDMD‐knockout vectors was achieved via retrograde pancreatic intraductal delivery to elucidate the role of GSDMD in macrophages. We administered either the HA‐tagged macrophage‐specific adeno‐associated viral vector (AAV9‐SP146‐C1‐GSDMD‐sgRNA) or a control vector (AAV9‐SP146‐C1‐GSDMD‐sgControl) to the AP mice (Figure S6A, Supporting Information). Notably, macrophages expressing the HA‐tag did not exhibit GSDMD staining, while acinar cells showed GSDMD staining, confirming the specific knockout of GSDMD in macrophages (Figure S6B, Supporting Information). Compared to the control group, mice receiving AAV9‐SP146‐C1‐GSDMD‐sgRNA demonstrated reduced serum inflammatory cytokine levels (Figure S6C, Supporting Information). However, no significant difference was observed in pancreatic enzyme levels between the two groups (Figure S6D, Supporting Information). In summary, utilizing the AAV‐mediated tissue‐specific knockout mouse model, we determined that GSDMD knockout in acinar cells enhanced pancreatic enzyme synthesis, whereas GSDMD knockout in macrophages diminished serum inflammatory cytokine levels.

### 2.4 GSDMD Reduces Pancreatic Enzyme Synthesis in Acinar Cells Partly through Inhibiting the AKT/mTOR/RPS6/4EBP1 Pathway

**Figure 4:**
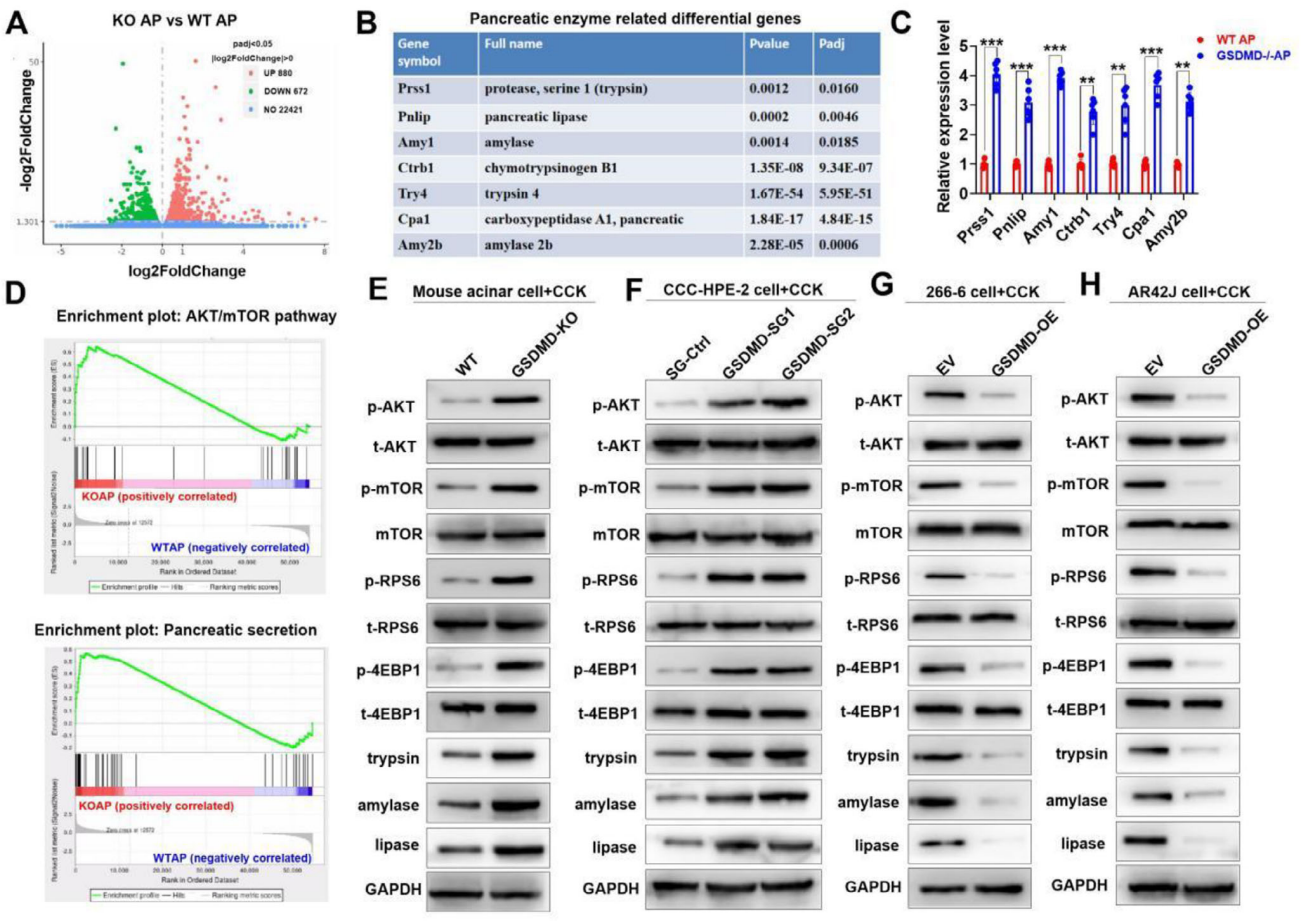
GSDMD reduces pancreatic enzyme synthesis in acinar cells partly through the inhibition of the AKT/mTOR/RPS6/4EBP1 pathway. A) RNA sequencing analysis of pancreatic tissues of the GSDMD^−/−^ AP (*n* = 3) and WT AP groups (*n* = 3). B) Pancreatic enzyme‐related differential genes are significantly expressed between the GSDMD^−/−^AP and WT AP groups. C) Expressions of pancreatic enzyme‐related differential genes were verified via RT‐PCR (*n* = 6 per group). D) GSEA showed that GSDMD knockdown enhanced the AKT/mTOR pathway and pancreatic secretion. E) Representative western blotting results of WT and GSDMD‐KO cholecystokinin‐stimulated (0.001 mm) primary cultured mouse acinar cells. F) Representative western blotting results of SG‐Ctrl and GSDMD‐SG cholecystokinin‐stimulated (0.001 mm) CCC‐HPE‐2 cells. G) Representative western blotting results of empty vector (EV) and GSDMD overexpressing (GSDMD‐OE) cholecystokinin‐stimulated (0.001 mm) 266‐6 cells. H) Representative western blotting results of EV and GSDMD‐OE cholecystokinin‐stimulated (0.001 mm) AR42J cells. Abbreviation: GSDMD, gasdermin D; CCK, cholecystokinin; EV, empty vector; OE, overexpressing; GSDMD‐SGs, single guide RNAs (sgRNAs) targeting GSDMD; SGCTR, control sgRNAs; WT, wild type; AP, acute pancreatitis; GSEA, gene set enrichment analysis. C) *p*‐values were determined by two‐tailed unpaired Student's *t*‐test. ***p < 0.001 and **p < 0.01.

Although the overall symptoms of acute pancreatitis in GSDMD^−/−^ mice were significantly relieved, serum trypsin, amylase, and lipase activities were higher than those in the control group. To further address these issues, we performed RNA sequencing of pancreatic tissues from the GSDMD^−/−^ AP and WT AP groups (**Figure** **4**A). Differential gene analysis showed that, compared to the WT AP group, the expressions of Prss1, Pnlip, and Amy1 genes encoding trypsin, lipase, and amylase, respectively, increased significantly in the GSDMD^−/−^ AP group (Figure 4B). We verified the RNA sequencing results using reverse transcription polymerase chain reaction (RT‐PCR) and western blotting (Figure 4C; Figure S7, Supporting Information), which further demonstrated that the GSDMD knockout promoted pancreatic enzyme synthesis by upregulating Prss1, Pnlip, and Amy1 expressions. Next, we explored the molecular pathways through which GSDMD inhibits the synthesis of pancreatic enzymes. Gene set enrichment analysis (GSEA) and Kyoto Encyclopedia of Genes and Genomes (KEGG) pathway analysis showed that GSDMD knockdown enhanced protein kinase B (AKT)/mammalian target of rapamycin (mTOR) pathway and pancreatic secretion (Figure 4D; Figure S8, Supporting Information). We used cholecystokinin (CCK) to stimulate acinar cells to establish a pancreatitis cell model, as previously reported.^[^ ^19^ ^]^ We found that AKT/mTOR/ribosomal protein S6 (RPS6)/eukaryotic translation initiation factor 4E‐binding protein 1 (4EBP1) signaling was activated in GSDMD‐knockout acinar cells but was inhibited in GSDMD‐overexpressing (GSDMD‐OE) acinar cells (Figure 4E–H). Moreover, overexpression of GSDMD effectively rescues the activation of the AKT/mTOR/RPS6/4EBP1 pathway induced by GSDMD‐KO (Figure S9, Supporting Information). In summary, our western blotting results showed that GSDMD reduced the synthesis of trypsin, amylase, and lipase, partly by inhibiting the AKT/mTOR/RPS6/4EBP1 pathway (Figure 4E–H).

### 2.5 GSDMD Mediates the Resistance of Pancreatic Acinar Cells to Digestive Enzymes

**Figure 5:**
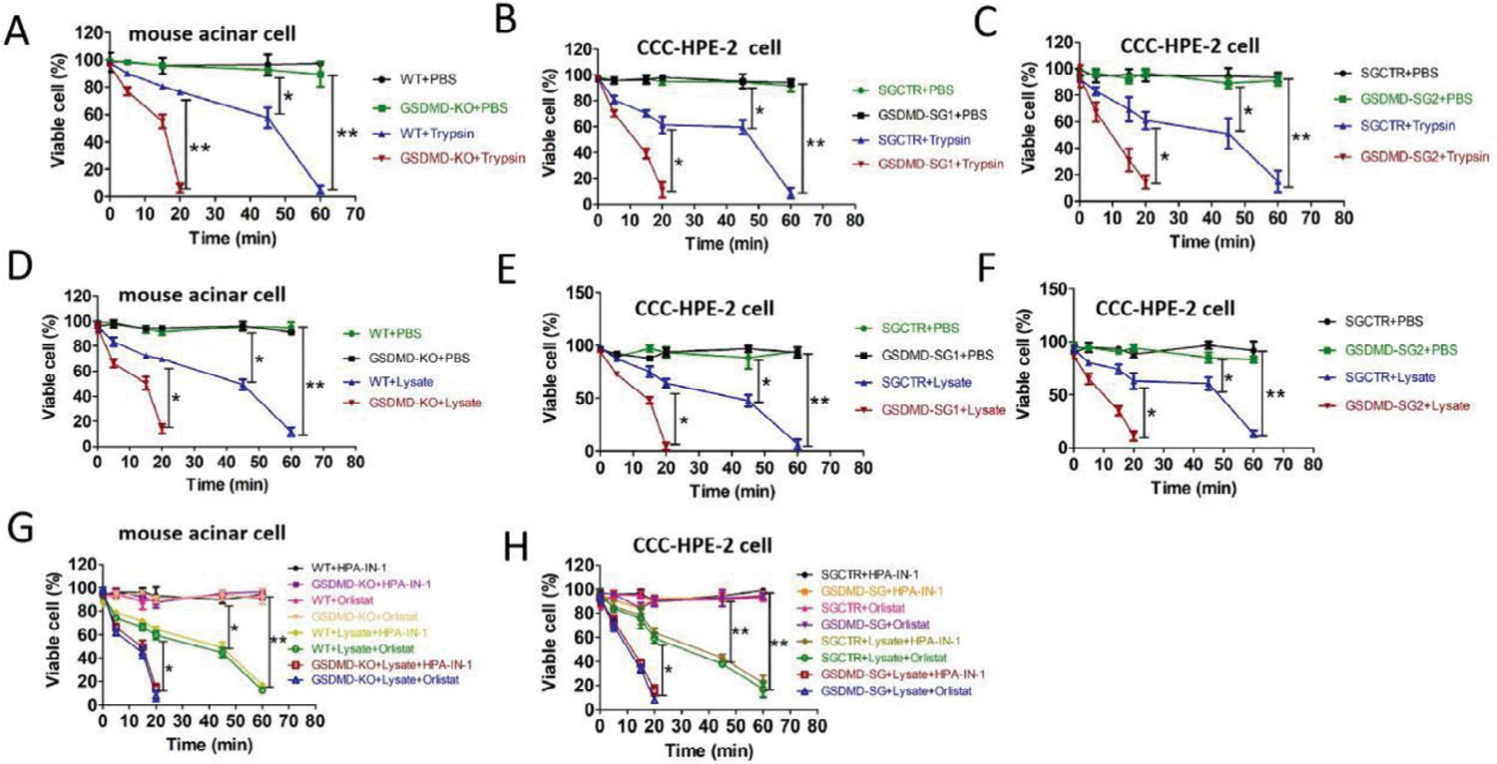
GSDMD helps the resistance of pancreatic acinar cells to trypsin‐mediated digestion. A) Primary cultured GSDMD‐knockout (KO) and wild‐type (WT) mouse pancreatic acinar cells were treated with trypsin (45 U mL^−l^). Cell viability was determined using ATP cell viability assay at 0, 5, 15, 20, 45, and 60 min. B,C) CCC‐HPE‐2 cells transfected with SGCTR or GSDMD‐SGs were treated with phosphate‐buffered saline (PBS) or trypsin (45 U mL^−1^). Cell viability was determined using ATP cell viability assay at 0, 5, 15, 20, 45, and 60 min. D) Primary cultured GSDMD‐KO and WT mouse pancreatic acinar cells were treated with lysate (20 µL mL^−1^) isolated from mouse pancreas. Cell viability was determined using ATP cell viability assay at 0, 5, 15, 20, 45, and 60 min. E,F) CCC‐HPE‐2 cells transfected with SGCTR or GSDMD‐SGs were treated with lysate (20 µL mL^−1^) isolated from mouse pancreas. Cell viability was determined using ATP cell viability assay at 0, 5, 15, 20, 45, and 60 min. G) Primary cultured GSDMD‐KO and WT mouse pancreatic acinar cells were treated with lysate (20 µL mL^−1^), human pancreatic α‐amylase inhibitor compound 1 (HPA‐IN‐1) (12 µm), lipase inhibitors Orlistat (12.5 µm), lysate + HPA‐IN‐1, or lysate + Orlistat. H) CCC‐HPE‐2 cells transfected with SGCTR or GSDMD‐SGs were treated with lysate (20 µL mL^−1^), amylase inhibitors HPA‐IN‐1 (12 µm), lipase inhibitors Orlistat (12.5 µm), lysate + HPA‐IN‐1, or lysate + Orlistat. Cell viability was determined using ATP cell viability assay at 0, 5, 15, 20, 45, and 60 min. Abbreviation: GSDMD, gasdermin D; GSDMD‐SGs, single guide RNAs (sgRNAs) targeting GSDMD; SGCTR, sgRNAs control. A–H) *p*‐values were determined by one‐way ANOVA with Dunnett's multiple comparisons. **p < 0.01 and *p < 0.1.

Because GSDMD can reduce pancreatic enzyme synthesis, we explored whether GSDMD could mediate the resistance of normal pancreatic acinar cells to digestive enzymes. Therefore, we treated primary cultured GSDMD‐KO and WT mouse pancreatic acinar cells with trypsin. We detected cell viability at different time points and found that GSDMD‐KO acinar cells showed a significant decrease in cell viability at 20 min, whereas WT acinar cells showed a significant decrease at 60 min, indicating that GSDMD deficiency significantly reduced pancreatic acinar cell resistance to pancreatic enzymes (**Figure** **5**A). To further confirm this result, we treated normal human pancreatic CCC‐HPE‐2 cells transfected with single guide RNAs (sgRNAs) targeting GSDMD (GSDMD‐SGs) with trypsin (Figure S10, Supporting Information). Trypsin caused a faster rate of cell death in cells transfected with GSDME‐SGs than in control cells (Figure 5B,C). Next, we treated primary cultured GSDMD‐KO and WT mouse pancreatic acinar cells with pancreatic lysates. The death rate of GSDMD‐KO acinar cells caused by the pancreatic lysate was significantly faster than that of WT acinar cells (Figure 5D). Similarly, pancreatic lysate caused a faster rate of cell death in CCC‐HPE‐2 cells transfected with GSDMD‐SGs than in the control cells (Figure 5E,F). In contrast, we found that the viability of WT cells significantly decreased at 60 min, whereas GSDMD‐OE cells did not show a significant decrease, indicating that GSDMD contributes to acinar cell resistance to trypsin (Figure S11, Supporting Information). Further, we treated primary cultured GSDMD‐OE and WT mouse acinar cells with pancreatic lysates. Similarly, the death rate of GSDMD‐OE cells caused by pancreatic lysate was significantly slower than that of WT cells (Figure S11, Supporting Information). Pancreatic enzymes mainly include trypsin, amylase, and lipase. Notably, the addition of amylase or lipase inhibitors did not affect the death rate of pancreatic lysate‐mediated GSDMD‐knockout cells (Figure 5G,H). This finding suggests that trypsin, instead of amylase and lipase, is involved in the rapid death of GSDMD‐knockout cells.

### 2.6 GSDMD Induces MUC1 Expression by Activating the NF‐κB Pathway, Thereby Increasing Resistance to Digestive Enzymes

**Figure 6:**
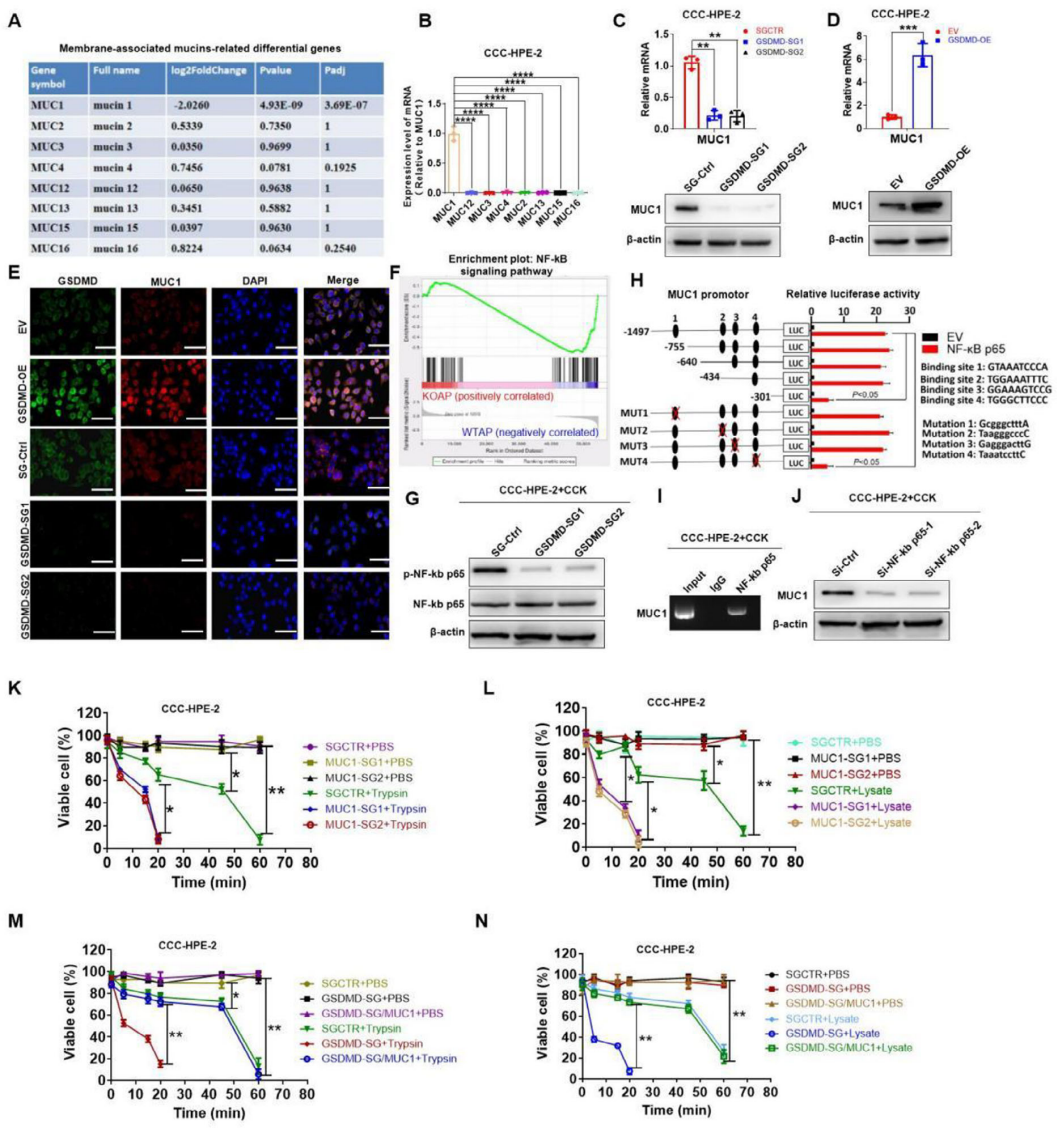
GSDMD induces the resistance of mucin 1 to digestive enzymes. A) Membrane‐associated mucin (MUC)‐related genes in the GSDMD^−/−^AP and WT AP groups. B) Messenger ribonucleic acid (mRNA) expression of MUCs (MUC1, MUC2, MUC3, MUC4, MUC12, MUC13, MUC15, and MUC16) from human pancreatic CCC‐HPE‐2 cells determined using RT‐PCR. C) The expression of MUC1 from SGCTR or GSDMD‐SGs‐CCC‐HPE‐2 cells determined using RT‐PCR and western blotting. D) The expression of MUC1 from EV or GSDMD‐overexpression‐CCC‐HPE‐2 cells determined using RT‐PCR and western blotting. E) Cellular immunofluorescence demonstrates changes in MUC1 following GSDMD interference or overexpression. Scale bars: 50 µm. F) GSEA and G) western blotting showed that a reduced effect of GSDMD knockout on NF‐κB signaling. H) The activity of serially truncated or mutated MUC1 promoter in cholecystokinin (CCK)‐stimulated (0.001 mm) CCC‐HPE‐2 cells detected via luciferase assay. I) Binding of NF‐κB p65 on MUC1 promotor in CCK‐stimulated CCC‐HPE‐2 cells was tested by ChIP assay. J) Representative western blotting results of siCtrl, sip65‐1, and si‐p65‐2 CCK‐stimulated (0.001 mm) CCC‐HPE‐2 cells. K) CCC‐HPE‐2 cells transfected with SGCTR or MUC1‐SGs were treated with PBS or trypsin (45 U mL^−1^). Cell viability was determined using ATP cell viability assay at 0, 5, 15, 20, 45, and 60 min. L) CCC‐HPE‐2 cells transfected with SGCTR or MUC1‐SGs were treated with PBS or lysate (20 µL mL^−1^) isolated from mouse pancreas. Cell viability was determined via ATP cell viability assay at 0, 5, 15, 20, 45, and 60 min. M) CCC‐HPE‐2 cells transfected with SGCTR, GSDME‐SG, or GSDME‐SG/MUC1 were treated with PBS or trypsin (45 U mL^−1^). Cell viability was determined using ATP cell viability assay at 0, 5, 15, 20, 45, and 60 min. N) CCC‐HPE‐2 cells transfected with SGCTR, GSDME‐SG, or GSDME‐SG/MUC1 were treated with PBS or lysate (20 µL mL^−1^) isolated from mouse pancreas. Cell viability was determined using ATP cell viability assay at 0, 5, 15, 20, 45, and 60 min. Abbreviation: GSDMD, gasdermin D; GSDMD‐SGs, single guide RNAs (sgRNAs) targeting GSDMD; MUC1‐SGs, sgRNAs targeting MUC1; SGCTR, sgRNAs control; GSEA, gene set enrichment analysis. D) *p*‐values were determined by two‐tailed unpaired Student's *t*‐test; B,C,H,K–N) one‐way ANOVA with Dunnett's multiple comparisons. ****p < 0.0001,***p < 0.001, **p < 0.01, and *p < 0.1.

We explored the specific mechanism of GSDMD‐mediated resistance of pancreatic cells to digestive enzymes. Because the mucosal epithelium protects itself against digestive enzymes by expressing a layer of mucosal molecules, we hypothesized that GSDMD promotes resistance to pancreatic enzymes by increasing the expression of membrane‐associated MUCs. We performed RNA sequencing analysis of pancreatic tissues from the GSDMD^−/−^ AP and WT AP groups. Differential gene analysis showed that, compared to the WT AP group, the expression of MUC1 decreased significantly in the GSDMD^−/−^ AP group. There were no significant differences in the expression levels of MUC2, MUC3, MUC4, MUC12, MUC13, MUC15, or MUC16 between the two groups (**Figure** **6**A). In addition, we detected the expression of MUCs in normal human pancreatic CCC‐HPE‐2 cells and found that the expression of MUC1 was relatively high (Figure 6B). In addition, GSDMD knockdown resulted in decreased MUC1 expression (Figure 6C), whereas GSDMD overexpression resulted in increased MUC1 expression (Figure 6D). Consistently, the cellular immunofluorescence results further demonstrated that GSDMD promotes the expression of MUC1 (Figure 6E). Further, we explored the molecular pathways through which GSDMD increases the expression of MUC1. GSEA and western blotting showed that GSDMD knockout weakened nuclear factor kappa‐B (NF‐κB signaling (Figure 6F,G; Figure S12, Supporting Information). NF‐κB p65 serves as a transcription factor that activates the expression of downstream genes. Our results showed that truncation of the MUC1 promoter from the −434 to −301 bp region or mutation of NF‐κB p65‐binding sequences in this region inhibited NF‐κB p65‐induced activation of MUC1 promoter (Figure 6H). Chromatin immunoprecipitation (ChIP) assay further verified the binding of the NF‐κB p65 to the MUC1 promoter (Figure 6I). Western blotting indicated that the expression of MUC1 decreased after interference with NF‐κB p65 (Figure 6J). These results indicate that GSDMD increases the expression of MUC1 through NF‐κB p65‐induced transcriptional activation of MUC1. Furthermore, we investigated whether GSDMD increases the resistance of acinar cells to digestive enzymes via MUC1. Our results showed that trypsin or pancreatic lysate caused a faster rate of cell death in cells transfected with MUC‐SGs than in control cells (Figure 6K,L). Moreover, GSDMD knockout shortened the resistance time of pancreatic cells to trypsin or pancreatic lysate, whereas MUC1 overexpression significantly prolonged the resistance time of pancreatic cells to trypsin or pancreatic lysate (Figure 6M,N). Taken together, these results suggest that GSDMD develops resistance to digestive enzymes by inducing the expression of MUC1 by pancreatic cells.

### 2.7 GSDMD Promotes the Expression of Inflammatory Cytokines in Macrophages

**Figure 7:**
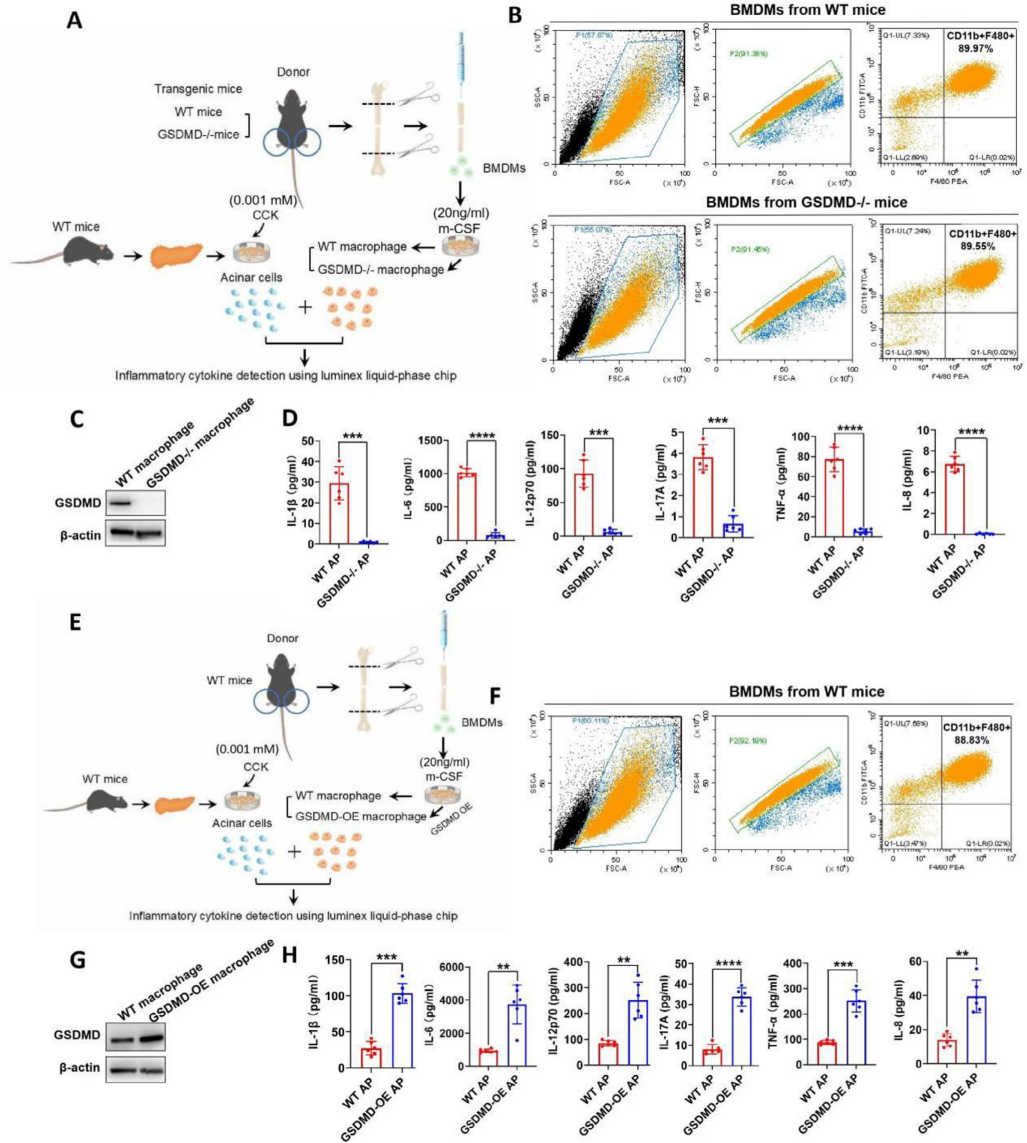
GSDMD promotes the expression of inflammatory cytokines in macrophages. A) Flow diagram of the experimental design to verify the effect of GSDMD knockout on macrophages. B) Flow cytometry confirmed the successful extraction of bone‐marrow‐derived macrophages (BMDMs) from WT and GSDMD^−/−^ mice. C) Immunoblotting analysis of the indicated proteins in WT macrophage and GSDMD^−/−^ macrophage. D) GSDMD^−/−^ macrophages or control macrophages were co‐incubated with freshly prepared CCK‐stimulated pancreatic acinar cells for 6 h, and the levels of inflammatory cytokines in the cell culture supernatant were detected. E) Flow diagram of the experimental design flow diagram to verify the effect of GSDMD overexpression on macrophages. F) Flow cytometry confirmed the successful extraction of BMDMs from WT mice. G) Immunoblotting analysis of the indicated proteins in WT and GSDMD‐overexpressing (GSDMD‐OE) macrophages. H) GSDMD‐OE or control macrophages were co‐incubated with freshly prepared CCK‐stimulated pancreatic acinar cells for 6 h, and the levels of inflammatory cytokines in the cell culture supernatant were detected. Abbreviation: GSDMD, gasdermin D; WT, wild type; AP, acute pancreatitis. D,H) *p*‐values were determined by two‐tailed unpaired Student's *t*‐test. ****p < 0.0001, ***p < 0.001, and **p < 0.01.

The infiltration of pancreatic macrophage was reduced in the GSDMD^−/−^ AP group compared to the WT AP group (Figure S13, Supporting Information). In addition, we investigated the role of GSDMD in macrophages (**Figure** **7**A). Bone‐marrow‐derived macrophages (BMDMs) were isolated from the bone marrow of WT and GSDMD^−/−^ mice. Flow cytometry confirmed the successful extraction of BMDMs (Figure 7B). 20 ng mL^−1^ macrophage colony‐stimulating factor (m‐CSF) was added to the medium to differentiate BMDMs into macrophages, as previously reported.^[^ ^19^ ^]^ Western blotting results confirmed minimal expression of GSDMD in GSDMD^−/−^ macrophages (Figure 7C). The pancreas from WT mice was digested by collagenase to prepare fresh acinar cells. Before co‐incubation with macrophages, the acinar cells were stimulated with 0.001 mm CCK for 30 min to simulate a cellular pancreatitis model.^[^ ^19^ ^]^ Thereafter, GSDMD^−/−^ or control macrophages were co‐incubated with freshly prepared pancreatic acinar cells for 6 h, and the levels of inflammatory cytokines in the cell culture supernatant were detected (Figure 7A). The levels of inflammatory cytokines (such as IL‐1β, IL‐6, IL‐12p70, IL‐17A, TNF‐α, and IL‐8) secreted by GSDMD^−/−^ macrophages were significantly reduced compared to the levels secreted by control macrophages (Figure 7D). Meanwhile, GSDMD‐OE macrophages secreted significantly higher levels of inflammatory cytokines than did control macrophages (Figure 7E–H). These results indicate that GSDMD promotes the secretion of inflammatory cytokines by macrophages in AP.

### 2.8 GSDMD Increases the Proportion of Peripheral Blood Th1 and Th17 Lymphocyte Subsets in Mice AP

**Figure 8:**
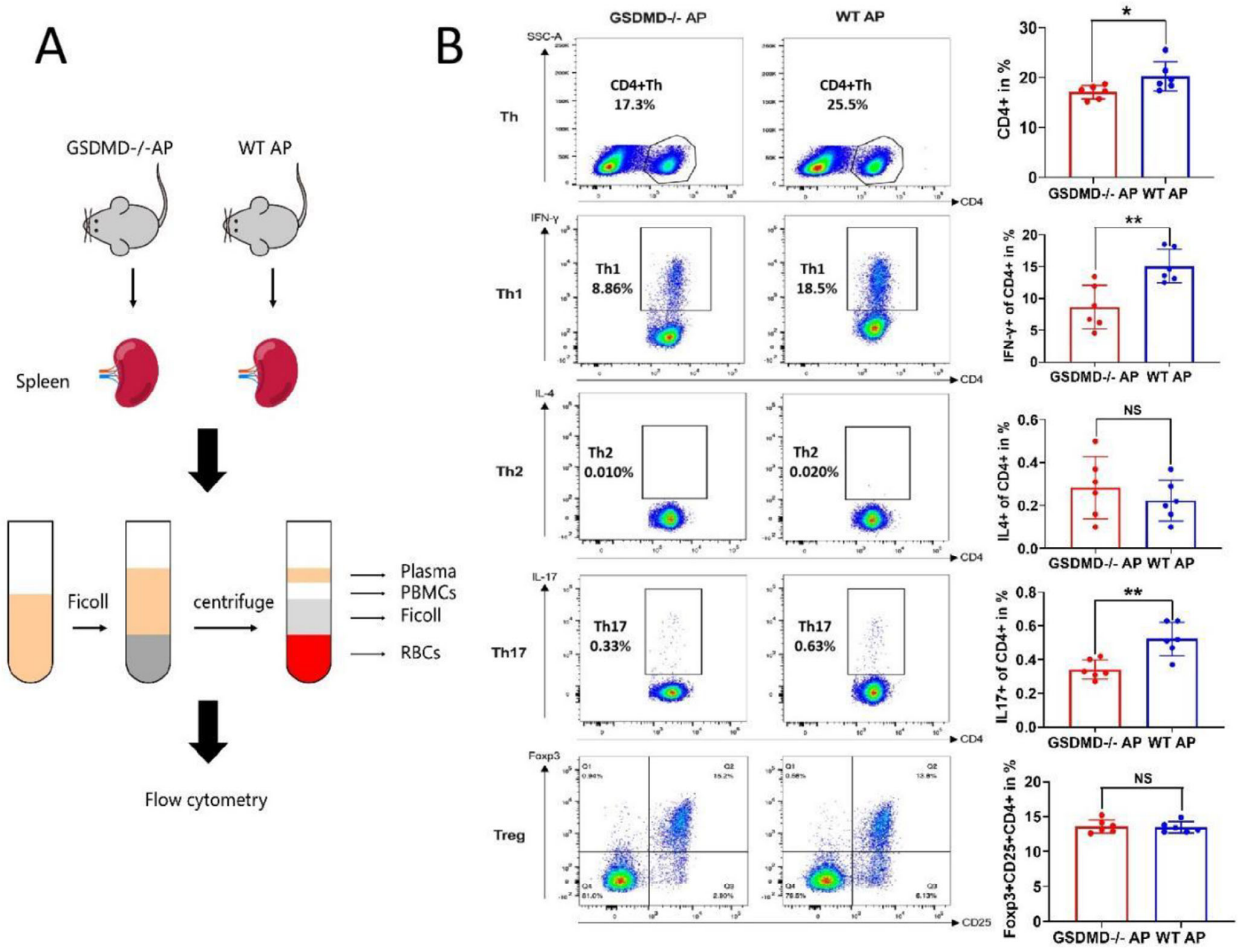
GSDMD increases the proportion of peripheral blood Th1 and Th17 lymphocyte subsets in mice acute pancreatitis. A) Flow cytometry detection process. Spleen single‐cell suspension of mice in the GSDMD^−/−^AP and WT AP groups was extracted to obtain peripheral blood lymphocytes, which was, thereafter, subjected to flow cytometry detection. B) The proportions of CD4^+^ Th, IFN‐γ^+^CD4^+^Th1, IL‐4^+^CD4^+^Th2, IL‐17^+^CD4^+^Th17, and Foxp3^+^CD25^+^CD4^+^Treg cells between the GSDMD^−/−^ AP (*n* = 6) and WT AP groups (*n* = 6) were analyzed using flow cytometry. Abbreviation: NS, not significant. WT, wild type; AP, acute pancreatitis; GSDMD, gasdermin D. B) *p*‐values were determined by two‐tailed unpaired Student's *t*‐test. **p < 0.01 and *p < 0.1.

We analyzed whether GSDMD, in addition to its role in the recruitment of macrophages, affects T lymphocyte subsets. Spleen single‐cell suspensions from mice in the GSDMD^−/−^ AP and WT AP groups were used to obtain peripheral blood lymphocytes. The proportion of CD4^+^ Th, interferon‐γ (IFN‐γ)^+^CD4^+^ Th1, IL‐4^+^CD4^+^ Th2, IL‐17^+^CD4^+^ Th17, and Foxp3^+^CD25^+^CD4^+^ regulatory T cells (Treg) cells between the two groups were analyzed using flow cytometry (**Figure** **8**A). The results showed that the proportions of helper T cells (Th), Th1, and Th17 cells in the peripheral blood of the GSDMD^−/−^ AP group were significantly lower than those in the peripheral blood of the WT AP group, whereas the proportions of Th2 and Treg cells were not significantly different (Figure 8B). These results suggest that GSDMD knockout reduces Th1‐ and Th17‐mediated inflammatory responses in AP.

## 3 Discussion

**Figure 9:**
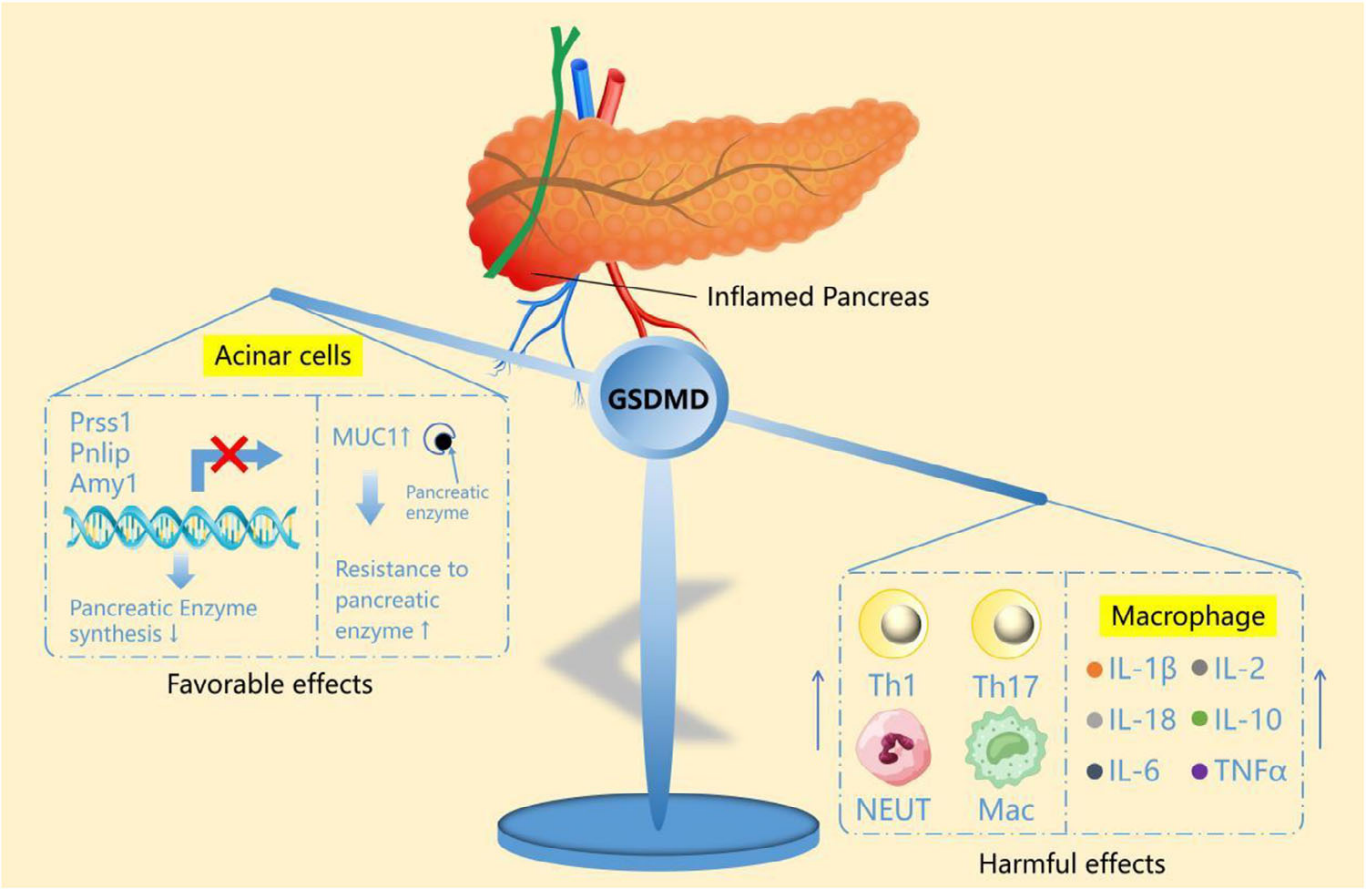
Schematic diagram showing that GSDMD plays two opposite roles in acute pancreatitis. GSDMD does not only reduce pancreatic enzyme synthesis but also induces pancreatic acinar cells to express MUC1, which forms a barrier to prevent digestive enzyme‐mediated digestion. However, GSDMD can promote the secretion of inflammatory cytokines by macrophages and aggravate pancreatic histological injury by expanding inflammatory response, which demonstrate the two opposite roles of GSDMD in acute pancreatitis. Abbreviation: GSDMD, gasdermin D.

The most important finding of this study is that GSDMD plays two different roles in relation to pancreatic acinar cells and macrophages in AP. In acinar cells, GSDMD inhibits pancreatic enzyme synthesis by downregulating the expressions of Prss1, Pnlip, and Amy1 via the inhibition of the AKT/mTOR/RPS6/4EBP1 pathway. Moreover, GSDMD induces pancreatic acinar cells to express MUC1 by activating the NF‐κB pathway, which forms a barrier preventing digestive enzyme‐mediated digestion. In contrast, GSDMD promotes the secretion of inflammatory cytokines by macrophages in AP. Furthermore, GSDMD increases the infiltration of macrophages and neutrophils in AP and increased the proportion of Th1 and Th17 lymphocyte subsets in peripheral blood. However, in general, the harmful effect of GSDMD in AP outweighs its beneficial effect, and GSDMD knockout can effectively alleviate AP (**Figure** **9**).

Studies have shown that GSDMD actively mediates the pathogenesis of inflammatory, infectious, and metabolic diseases. For example, nonalcoholic steatohepatitis,^[^ ^9^ ^]^ inflammatory bowel disease,^[^ ^20^ ^]^ and rheumatoid arthritis^[^ ^21^ ^]^ are closely associated with GSDMD. In addition, GSDMD is a potential target for infectious diseases, such as bacillary dysentery^[^ ^22^ ^]^ and tuberculosis.^[^ ^23^ ^]^ To investigate whether GSDMD is involved in the progression of AP, GSDMD^−/−^ mice and WT littermates were injected intraperitoneally with caerulein to establish an AP model. After AP establishment, the pancreas of the WT group showed more severe edema and inflammatory infiltration than that of the GSDMD^−/−^AP group. Consistently, the levels of serum inflammatory factors such as IL‐1β, IL‐18, IL‐6, IL‐10, IL‐12p70, and TNF‐α in the GSDMD^−/−^ AP group were lower than those in the WT AP group, suggesting that GSDMD may play a role in promoting inflammation in AP. Notably, IL‐10 is an anti‐inflammatory cytokine that inhibits the secretion of pro‐inflammatory cytokines by monocytes and macrophages.^[^ ^24^ ^]^ Previous studies have shown the elevation of IL‐10 in the serum of patients with AP to inhibit the excessive secretion of pro‐inflammatory cytokines, which reflects a protective mechanism of the body.^[^ ^25^, ^26^, ^27^ ^]^ Therefore, we speculate that GSDMD knockout reduces the release of pro‐inflammatory cytokines, leading to the feedback downregulation of IL‐10 expression.

In addition, to determine whether GSDMD affects inflammatory response by its effects on, we isolated BMDMs from WT and GSDMD^−/−^ mice. Macrophage colony‐stimulating factor was added to the medium to differentiate BMDMs from macrophages, as previously reported.^[^ ^19^ ^]^ Fresh acinar cells were prepared from the pancreas of WT mice using collagenase digestion. Before co‐incubation with macrophages, the acinar cells were stimulated with CCK to simulate a cellular pancreatitis model.^[^ ^19^ ^]^ Thereafter, GSDMD^−/−^ or control macrophages were co‐incubated with freshly prepared pancreatic acinar cells, and the levels of inflammatory cytokines in the cell culture supernatant were measured. Our results showed that the levels of inflammatory cytokines (IL‐1 β, IL‐6, IL‐12p70, IL‐17A, TNF‐α, and IL‐8) secreted by GSDMD^−/−^ macrophages were significantly reduced compared to those secreted by control macrophages. In contrast, GSDMD‐OE macrophages secreted significantly higher levels of inflammatory cytokines than did the control macrophages. These results indicated that GSDMD promotes the secretion of inflammatory cytokines by macrophages in AP.

GSDMD not only promotes the secretion of inflammatory cytokines by macrophages in AP but also affects various immune cells. Our results showed that the expressions of multiple chemokines, such as IL‐8, CCL2, CCL3, and CCL5, were downregulated in the GSDMD^−/−^ AP group compared to the WT AP group. These chemokines are involved in the recruitment and migration of neutrophils and macrophages to injured tissue.^[^ ^28^, ^29^, ^30^ ^]^ Consistent with this, the infiltration of macrophages and neutrophils in the pancreas of the GSDMD^−/−^ AP group was reduced compared to that in the WT AP group. Th cells are a major class of CD4^+^T cells. Activated Th cells regulate the survival, development, and differentiation of other types of immune cells, such as Th1, Th2, Th9, Th17, and Tfh cells, via several mechanisms.^[^ ^31^ ^]^ An increase in the Th1 subgroup was detected in local tissue and peripheral blood in AP.^[^ ^32^ ^]^ Moreover, Th1‐mediated immune responses and related cytokines are associated with AP severity.^[^ ^33^ ^]^ Th17 cells induce pancreatic acinar cell necrosis by secreting IL‐17A,^[^ ^34^ ^]^ which is valuable for evaluating AP prognosis.^[^ ^35^ ^]^ Our results revealed that the proportions of Th1 cells and Th17 cells in peripheral blood of the GSDMD^−/−^ AP group were significantly lower than that of the WT AP group, suggesting that GSDMD knockout can reduce Th1‐ and Th17‐mediated inflammatory response in AP.

Notably, although GSDMD promoted inflammatory responses, it inhibited the synthesis of pancreatic enzyme. The levels of serum trypsin, amylase, and lipase in the GSDMD^−/−^ AP group were significantly higher than those in the WT AP group, suggesting that GSDMD knockout increased pancreatic enzyme synthesis in AP. Furthermore, GSEA showed that GSDMD knockdown enhanced the AKT/mTOR pathway and pancreatic secretion. Moreover, GSDMD knockout increased the expression of Prss1, Pnlip, and Amy1, which encode trypsin, lipase, and amylase, respectively.^[^ ^36^, ^37^, ^38^ ^]^ This result indicated that GSDMD inhibits pancreatic enzyme synthesis by downregulating the expression of Prss1, Pnlip, and Amy1. Mechanistically, we found that AKT/mTOR/RPS6/4EBP1 signaling was activated in GSDMD‐knockout acinar cells but was inhibited in GSDMD‐OE acinar cells. Previous studies have shown that mTORC promotes protein synthesis via RPS6 and 4EBP1 downstream cascade.^[^ ^39^ ^]^ It is known that the phosphorylation of 4EBP1 inhibits its ability to bind to eIF4E, thereby releasing eIF4E to participate in the assembly of the eIF4F complex which is required for the initiation of translation process.^[^ ^40^ ^]^ Our results demonstrated that GSDMD knockout increases the synthesis of trypsin, amylase, and lipase, partly by activating the AKT/mTOR/RPS6/4EBP1 pathway. However, our results contradict the findings of previous studies showing the inhibition of pancreatic enzyme secretion after GSDMD knockout. Careful examination revealed that our experimental conditions differed from those reported in the literature. In our study, a MAP mouse model was used, and each mouse was intraperitoneally injected with 50 µg kg^−1^ caerulein once per hour for a total of eight injections. However, Wu et al. used a SAP mouse model, which was intraperitoneally injected with caerulein (50 µg kg^−1^) every hour for a total of seven times. At the last injection of caerulein, the mice received an LPS (10 mg kg^−1^, intraperitoneal injection (i.p.)) injection.^[^ ^12^ ^]^ Besides, Gao et al. used a SAP mouse model, which was intraperitoneally injected with caerulein (200 µg kg^−1^) every hour, for a total of ten times.^[^ ^13^ ^]^ We hypothesized that in MAP, GSDMD‐knockout acinar cells remain functional, activating the AKT/mTOR/RPS6/4EBP1 pathway to promote the secretion of pancreatic enzymes. However, in SAP, most GSDMD‐knockout acinar cells become necrotic and cannot synthesize pancreatic enzymes, resulting in decreased pancreatic enzyme levels. Some clinical studies have shown that pancreatic tissues in patients with SAP may undergo extensive necrosis, leading to decreased pancreatic enzyme levels.^[^ ^41^, ^42^ ^]^ Therefore, pancreatic enzyme levels are associated with disease severity and stage. However, the mechanism of action of GSDMD in SAP remains unclear and requires further investigation.

Notably, our results revealed the GSDMD‐mediated resistance of pancreatic acinar cells to digestive enzymes. GSDMD knockout significantly accelerated the death rate of normal acinar cells treated with trypsin or pancreatic lysates. Pancreatic exocrine enzymes include trypsins, amylases, and lipases.^[^ ^43^ ^]^ Previous studies have shown that trypsin can cause cell death by breaking down cell membrane proteins.^[^ ^44^ ^]^ Trypsin also induces inflammation and oxidative stress, leading to cell death. However, the cytotoxic effects of amylase and lipase are weak and usually do not directly damage the cell structure.^[^ ^44^ ^]^ In addition, our results showed that amylase and lipase were not involved in the rapid death of GSDMD‐knockout cells caused by pancreatic lysate. In addition, we speculate that GSDMD knockout play in role in trypsin‐induced cell death but not in amylase‐ or lipase‐induced death because amylase and lipase are produced in inactive forms in the pancreas and activated by trypsin.

Since the mucosal epithelium protects itself against digestive enzymes by expressing a layer of mucosal molecules,^[^ ^45^, ^46^ ^]^ we hypothesized that GSDMD promotes resistance to pancreatic enzymes by increasing mucin expression. Our results demonstrate that GSDMD promoted MUC1 expression. Furthermore, we explored the molecular pathways through which GSDMD increases the expression of MUC1. GSEA and western blotting showed that GSDMD knockout weakened NF‐κB signaling. NF‐κB p65 serves as a transcription factor that activates the expression of downstream genes. Our results showed that the truncation of the MUC1 promoter or the mutation of NF‐κB p65‐binding sequences inhibited NF‐κB p65‐induced activation of MUC1 promoter. Furthermore, using ChIP assay, we verified the binding of the NF‐κB p65 to the MUC1 promoter. These results indicate that GSDMD increases the expression of MUC1 through the NF‐κB p65‐induced transcriptional activation of MUC1.

A very significant result of our study is that GSDMD knockout shortened the resistance time of pancreatic cells to trypsin or pancreatic lysate, whereas MUC1 overexpression significantly prolonged the resistance time of pancreatic cells to trypsin or pancreatic lysate. Taken together, these results suggest that GSDMD develops resistance to digestive enzymes by inducing pancreatic cells to express MUC1.

In addition, our study demonstrated the complex and opposite roles of GSDMD in AP. Using a tissue‐specific knockout model, we found that GSDMD plays different roles in pancreatic acinar cells and macrophages. GSDMD inhibition can activate the AKT/mTOR/RPS6/4EBP1 pathway to promote the synthesis of trypsin, amylase, and lipase in acinar cells. The secretion of pancreatic enzymes can promote injury, and damaged pancreatic cells can exacerbate the inflammatory response. However, the levels of inflammatory cytokines (IL‐1 β, IL‐6, IL‐12p70, IL‐17A, TNF‐α, and IL‐8) secreted by GSDMD^−/−^ macrophages were significantly reduced compared to those secreted by control macrophages. Notwithstanding, we found that, compared with WT AP mice, GSDMD^−/−^ AP mice exhibited reduced pancreatic injury, decreased serum inflammatory cytokines, decreased infiltration of pancreatic tissue by inflammatory cells, and decreased proportions of total Th cells, Th1 cells, and Th17 cells in peripheral blood. This result suggests that the pro‐inflammatory response mediated by pancreatic enzyme secretion caused by GSDMD knockout in acinar cells may be weaker than the anti‐inflammatory response caused by GSDMD knockout in macrophages. Therefore, GSDMD may be a potential target for the treatment of AP; however, its opposite effects need to be comprehensively considered, especially the complex relationship between pancreatic cell damage and inflammatory response.

Although this study provides new insights into the role of GSDMD in AP, it has some limitations. First, the experiments were conducted using murine models, which may not fully mimic the microenvironment of humans AP. Second, although we revealed an important role for GSDMD in AP, the molecular mechanisms underlying the interactions between GSDMD and other inflammatory pathways require further investigation. Third, the relationship and mechanism between the pro‐inflammatory response caused by pancreatic enzyme secretion due to GSDMD knockout in acinar cells and the anti‐inflammatory response caused by GSDMD knockout in macrophages remain unclear. Finally, this study focused on the role of GSDMD in MAP and did not evaluate its effect on the development of SAP.

In conclusion, we found that GSDMD plays opposing roles in AP. Regarding the positive, GSDMD inhibits pancreatic enzyme synthesis and develops resistance to digestive enzymes by inducing the expression of MUC1 by pancreatic acinar cells. In contrast, GSDMD promotes the secretion of inflammatory cytokines by macrophages in AP. Meanwhile, GSDMD increased the infiltration of macrophages and neutrophils in the pancreas and increased the proportion of Th1 and Th17 lymphocyte subsets in peripheral blood. Therefore, GSDMD may be a potential target for treating AP. However, its two opposing roles need to be comprehensively considered.

## 4 Experimental Section

Additional detailed materials and methods are included in the Supporting Information.

### Human Participants

Patients in the Department of Colorectal Surgery, The First Affiliated Hospital of Zhejiang University, between August 2020 and August 2023, were included in the study. According to the revised Atlanta Classification of Acute Pancreatitis, AP severity was classified into MAP, MSAP, and SAP.^[^ ^47^ ^]^ Fifteen patients with MAP, 15 with MSAP, 30 with SAP, and 30 healthy controls were enrolled in the study. All participants signed informed consent forms in accordance with the requirements of the Clinical Research Ethics Committee of The First Affiliated Hospital of Zhejiang University. All the programs complied with the ethical guidelines of the 1975 Declaration of Helsinki.

### Animals

GSDMD^−/−^ mice used in this study were a generous gift from Prof. Feng Shao (National Institute of Biological Sciences, Beijing, China). To construct GSDMD^−/−^ mice, Cas9 mRNA and guide RNA (gRNA) (5′AGCTACCTGGCATTCCGAG‐3′) were injected into the fertilized eggs of C57BL/6NCrl mice, and the T7E1 method was used to detect the accuracy of the insertion sequence. This mutation causes a 37 bp deletion in exon 5 of the mouse GSDMD gene, resulting in a frameshift mutation that inactivates the GSDMD protein. This study was approved by the Animal Research Ethics Committee of The First Affiliated Hospital of Zhejiang University.

### Induction of AP

In the AP group, each mouse was intraperitoneally injected with 50 µg kg^−1^ of caerulein, once every hour, for a total of eight injections. The control group mice were injected with an equal amount of physiological saline, once every hour, for a total of eight times. Four hours after the last injection, the mice were euthanized and their pancreas was collected. Some samples were placed in 5 mL of 4% paraformaldehyde, whereas others were frozen in liquid nitrogen to prepare for the next step of protein extraction.

### Immunohistochemical Staining

The paraffin slices were baked in the oven at 60 °C for 30 min, dewaxed in xylenes, 100% ethanol and 95% ethanol successively, and then placed in 0.01 m citric acid buffer for antigen repair. After sealing with 5% goat serum, the slices were incubated with GSDMD antibody (1:200, Cat. No. ab219800, Abcam, Cambridge, UK), F4/80 antibody (1:200, Cat. No. ab6640, Abcam, Cambridge, UK), and MPO antibody (1:200, Cat. No. ab208670, Abcam, Cambridge, UK) and then incubated at 4 °C overnight. The next day, the second antibody was added for incubation, and 200 µL diaminobenzidine (DAB) solution was added for color development.

### Western Blotting Assays

Western blotting was performed, as previously described.^[^ ^48^, ^49^ ^]^ Briefly, 20 µg of total protein per lane was separated using ‌sodium dodecyl sulfate polyacrylamide gel electrophoresis (‌SDS‐PAGE) and then transferred to a polyvinylidene fluoride (PVDF) membrane, which were blocked with 5% milk for 1 h and incubated with GSDMD antibody (1:1000, Cat. No. ab219800, Abcam, Cambridge, UK), caspase‐1 antibody (1:2000, Cat. No. ab138483, Abcam, Cambridge, UK), caspase‐11 antibody (1:2000, Cat. No. ab246496, Abcam, Cambridge, UK), and glyceraldehyde‐3‐phosphate dehydrogenase (GAPDH) antibody (1:2000, Cat. No. ab9485, Abcam, Cambridge, UK) at 4 °C overnight. The secondary antibody was incubated for 1 h on the following day. Signals were detected using an hypersensitive chemiluminescence (ECL) kit (Pierce, Rockford, IL, USA).

### Cell Lines

The CCC‐HPE‐2 cell line derived from human embryonic pancreatic tissue was purchased from the China Center for Type Culture Collection. CCC‐HPE‐2 cells were routinely cultured in Dulbecco's Modified Eagle Medium (DMEM) supplemented with 20% fetal bovine serum (FBS) (Gibco; Thermo Fisher Scientific). Pancreatic cells derived from primary culture of GSDMD^−/−^ mouse pancreatic tissue were cultured in DMEM with 20% FBS (Gibco; Thermo Fisher Scientific). No evidence of mycoplasma contamination was found using PCR‐based assays.

### Cell Viability Detection

CellTiter‐Glo Luminescent Cell viability Assay Kit (Promega) was used to detect cell viability at different time points after the addition of trypsin or pancreatic lysate. This kit is based on a luciferase reaction and measures the amount of ATP in live cells, which is closely related to cell viability.

### RNA Sequencing

RNA sequencing was performed, as described previously.^[^ ^50^ ^]^ Briefly, total RNA was extracted from pancreatic samples using a TRIzol kit (Promega, USA). The complementary DNA (cDNA) fragments were purified and enriched via PCR to construct the final cDNA library and sequenced on the Illumina sequencing platform (IlluminaHiSeq2500). Quality inspection results of sequencing data were shown in Table S1 (Supporting Information). The primers used for RT‐PCR are listed in Table S2 (Supporting Information). Representative dysregulated genes between the GSDMD^−/−^ AP and WT AP groups are shown in Table S3 (Supporting Information).

### Bioinformatics Analysis

The GSE194331 dataset, consisting of RNA‐seq data from 32 healthy controls and 87 AP blood samples, was downloaded from the Gene Expression Synthesis Database (https://www.ncbi.nlm.nih.gov/geo/). Using this dataset, the differences in serum expressions of IL‐1β, IL‐18, caspase 1, and GSDMD were analyzed between patients with AP and healthy controls, and the relationship between the levels of IL‐1β, IL‐18, caspase 1, and GSDMD and AP severity was analyzed.

### Statistical Analysis

All statistical analyses were performed using GraphPad Prism 10.0 (GraphPad Software, Inc., La Jolla, CA). Data were expressed as the mean ± standard deviation (mean ± SD). The data from the two unpaired groups were compared using a two‐tailed unpaired Student's *t*‐test. Statistical comparisons among three or more data groups were performed via one‐way ANOVA followed by Dunnett's multiple comparison test, and Pearson's correlation analysis was used to evaluate the correlation. A *p*‐value of <0.05 was considered statistically significant.

## Conflict of Interest

The authors declare no conflict of interest.

## Author Contributions

C.X.L., N.L., C.W., Z.Q. contributed equally to this work. C.X.L. and N.L. conceived and designed the study. C.W., Z.Y.Q., Q.H., X.L.W., S.R.Z., and S.H.L. performed the experiments and acquired and analyzed the data. C.X.L., G.W., T.Y., and N.L. were responsible for project administration and collection of clinical data. Q.W. and C.X.L. drafted and critically revised the manuscript.

## Supporting information



Supporting Information

## Data Availability

The data that support the findings of this study are available in the supplementary material of this article.
