# Supplementary material for: Gasdermin D‐Mediated Pyroptosis Exerts Two Opposite Effects of Resisting Enzymatic Digestion and Expanding Inflammatory Response in Acute Pancreatitis
Source: Adv Sci (Weinh). 2025 May 29;12(31):e02412. doi: 10.1002/advs.202502412 (PMC12376566; doi:10.1002/advs.202502412)
Supplement: Supplementary file 1 — Supporting Information [file ADVS-12-e02412-s001.docx]

Supporting Information

**Gasdermin D-mediated pyroptosis exerts two opposite effects of resisting enzymatic digestion and expanding inflammatory response in acute pancreatitis**

*Chaoxu Liu*^#,^*, *Ning Liang*^#^, *Chen Wu*^#^, *Zhaoyan Qiu*^#^, *Qian Huang*, *Xiaolong Wei*, *Shurong Zhang*, *Shuanghong Lei*, *Tao Yang**, *Gang Wang**, *Qian Wang**

C. X. Liu, N. Liang, C. Wu, and Z.Y. Qiu share co-first authorship.

*Correspondence Authors. Qian Wang, E-mail: wangqian1187@fmmu.edu.cn; Gang Wang, E-mail: [docwongg9@fmmu.edu.cn;](mailto:wg7995@126.com;) Chaoxu Liu, E-mail: [1520003@zju.edu.cn](mailto:1520003@zju.edu.cn); Tao Yang, E-mail: ttjrkyt@fmmu.edu.cn.

This file includes:

**Supplementary Experimental Section**

Figure S1. GSDMD-mediated pyroptosis is activated in mouse models of acute pancreatitis.

Figure S2. Western blotting analysis of GSDMD expression in different tissues from WT and KO mice.

Figure S3. There was no significant difference in pancreatic pathological morphology and inflammatory indexes between GSDMD-/- and wild-type control mice.

Figure S4. GSDMD knockout reduced the pathological score of mice with pancreatitis.

Figure S5. GSDMD knockout in acular cells increased the synthesis of pancreatic enzymes *in vivo*.

Figure S6. GSDMD knockout in macrophages reduced the levels of serum inflammatory cytokines *in vivo*.

Figure S7. The expression of pancreatic enzyme-related differential genes in GSDMD-/- AP and WT AP mice were verified via Western blotting.

Figure S8. The pancreatic synthesis and inflammation-related pathways were significantly enriched in the GSDMD -/- AP group.

Figure S9. Representative Western blotting results of WT, GSDMD-KO and GSDMD-KO+GSDMD-OE cholecystokinin (CCK)-stimulated (0.001 mM) primary cultured mouse acinar cells.

Figure S10. Western blotting results, showing sgRNA knockout of GSDMD in CCC-HPE2 cells.

Figure S11. GSDMD contributes to the resistance of acinar cells to trypsin.

Figure S12. GSDMD knockout weakens NF-κB signaling.

Figure S13. GSDMD increases the infiltration of macrophages and neutrophils in acute pancreatitis.

Table S1. Quality inspection results of sequencing data.

Table S2. Sequences of primers.

Table S3. List of representative dysregulated genes between the GSDMD-/- AP and WT AP groups.

**Supplementary Experimental Section**

***Identification of mouse genotypes***

Methods for the identification of mouse genotypes were as follows: scissors and tweezers were sterilized with 70% ethanol, and the tail tip of the mice was cut out by 0.2 cm and put in a 1.5 ml centrifuge tube. According to the description of the rat tail genotype identification kit (Cat. No. D7283M, Beyotime, Shanghai, China), 100 μL digestive fluid was added and heated in a 55 ℃ water bath for 15 min, and the sample was incubated in a 95 ℃ water bath for 5 min; 100 μL stop solution was added, and the product was directly used as a sample for further PCR experiment. The GSDMD primer sequences were as follows:

Forward primer (F1): 5 '- CACCCCTGAACTACC-3';

Reverse primer (R1): 5 '- GCACTCACCCCATTTAGAGC-3'.

PCR conditions were as follows: initial denaturation at 94 ℃ for 3 min, denaturation at 94 ℃ for 30 s, annealing at 55 ℃ for 30 s, extension at 72 ℃ for 20 s, 30 cycles; Extend at 72 ℃ for 10 min and store at 4 ℃. Thereafter, DNA agarose gel electrophoresis was performed. GSDMD-/- mice showed a 102 bp band, heterozygotes showed two bands of 120 bp and 140 bp, and the wild-type mice showed a 140 bp band.

***Flow cytometry analysis of T cells in mouse spleen***

1. Preparation of spleen single-cell suspension: After isolating the mouse spleen, it was placed on a 70 μ m cell filter and grinded evenly. The ground spleen was rinsed slowly with pre-cooled PBS to obtain spleen cell suspension. One milliliter of erythrocyte lysate was added to the cell suspension and shaken for 1 min. The resulting solution was centrifuged at 500 rpm for 10 min at 4℃, the supernatant was discarded; the resulting sample was washed with pre-cooled PBS twice, re-suspended in RPMI1640 medium containing 10% fetal bovine serum. The cell concentration was adjusted to 2×10^6^ cells /ml.
2. Stimulus: 2 ml of freshly prepared spleen single-cell suspension was combined with appropriate amounts of phorbol ester and ionomycin for stimulation, resulting in final concentrations of 25ng/ml and 1 μ g/ml. An appropriate amount of monensin was added to the block to achieve a final concentration of 1.7 μ g/ml. After thorough mixing, the mixture was placed in a cell culture incubator containing 5% CO2 at 37 ℃ for 6 h for stimulation. After stimulation, the sample was centrifuged at 500 rpm for 10 min at 4 ℃, washed with pre-cooled PBS, and resuspend in RPMI1640 medium containing 10% fetal bovine serum.
3. Blocking of Fc receptors: Anti-mouse CD16/CD32 antibodies were added at a dose of 100 μL per 10^6^ cells and incubated on ice for 5 min to block the Fc receptor.
4. Surface antibody labeling: 100 μL unstimulated spleen single-cell suspension was taken from A tube, and 0.5μl CD4-BV421, CD25-APC, CD69-FITC antibodies were added to the suspension. In addition, 100 μL stimulated spleen single-cell suspension was taken from B tube and 0.5μl CD4-FITC antibody was added. The mixture was properly mixed and incubated at 4 ℃ for 30 min in the dark.
5. Membrane rupture: 100 μ l of fixed membrane rupture solution was added to each tube; the resulting solution was properly mixed and incubate at 4 ℃ in the dark for 20 min.
6. Intracellular antibody labeling: 1μl Foxp3-PE antibody was added to tube A; 1 μL IFN-γ-BV421, IL-4-APC, and IL-17-PE antibodies were added to tube B, mixed, and incubated at 4 °C for 30 min without light. After incubation, appropriate amount of pre-cooled PBS solution was added, centrifuged and washed twice, and suspended with 500 μL PBS solution.
7. The detection was performed using a BD Accuri C6 flow cytometer (BD Biosciences).

***Luminex liquid phase chip for detecting serum inflammatory factors***

1. Sample preparation: After blood was taken through cardiac puncture, it was coagulated at room temperature for 2 h, centrifuged at 13000 rpm at 4℃ for 20 min, a supernatant was taken, and then transferred to the refrigerator for storage at -80 ℃.
2. Preparation of standard, reference, and serum matrix: According to the instructions of the reagent kit, 25 μL Matrix-11 was added to the standard bottle, vortexed for 10 s, and thoroughly mixed to obtain the Reconstituted Standard dilution standard curve. Thereafter, 250 μL Matrix-11 was added to Control-1 and Control-2, according to the instructions, vortexed, and properly mixed. Afterward, 2 ml assay buffer was added to the Serum Matrix, according to the manufacturer’s instructions, and vortexed for 30 s.
3. Sample incubation: magnetic beads were taken and oscillated at 1400 rpm for 30 s on the oscillator, and 25 μL per well was added to the 96-well plate. A 25 μL sample was added to the corresponding well and a 25 μL assay buffer was added. Thereafter, another 50 μL prepared standard, Blank, and Control were added into the corresponding hole; a sealing film was placed on the hole, placed it on a flat plate oscillator for shock at 850 rpm, placed in the dark, and incubated overnight at 4 ℃.
4. Antibody incubation: The sample was discarded, washed three times using a washing machine, 25 μL of Detection Antibody was added to each well, a sealing film was attached, and the plate shaker was used at 850 rpm in the dark for 1 h at room temperature.
5. Color development: 25 μL of Streptavidin PE was added to each well, the plate shaker was used at 850 rpm in the dark for 30 min, 150 μL of Sheath Fluid resuspended microbeads was added to each well, and the plate shaker was used at 850 rpm in the dark for 5 min. The wells were inserted into a calibrated Bio Flex instrument to read the values.

***Serum amylase and lipase detection***

Serum amylase and lipase activities were determined using a fully automated biochemical analyzer at the Laboratory Department of The First Affiliated Hospital of Zhejiang University.

***Pancreatic trypsin activity***

Pancreatic trypsin activity was detected, as previously described [1, 2]. In short, pancreatic tissue was homogenized in a cold buffer and the supernatant was collected after centrifugation. The sample supernatant was tested with the reaction buffer for 10 min using a flat plate reader (CLARIOstar, BMG Labtech) at an excitation wavelength of 380 nm and an emission wavelength of 440 nm. Standard curves were generated using purified human trypsin. Finally, pancreatic trypsin activity was detected using BCA protein assay (P0012, Bayotay).

***Pancreatic MPO activity***

Substrate 3,3',5,5'-tetramethylbenzidine (TMB, Sigma-Aldrich, 54827-17-7) was used to detect pancreatic MPO activity, as previously described [1, 2]. Briefly, pancreatic tissue was homogenized in a cold buffer and the supernatant was collected after centrifugation. The supernatant was mixed with phosphate buffer. The mixture was incubated at 37 °C for 3 min, and 0.01% hydrogen peroxide was added. Standard curves were generated using purified human MPO.

***H&E staining***

The paraffin slices were baked in the oven at 60 ℃ for 30 min, then dewaxed in xylenes, 100% ethanol and 95% ethanol, successively. The sections were stained with Harris hematoxylin for 5 min and then rinsed slowly with ddH_2_O for 5 min. The samples were soaked in 1% hydrochloric acid alcohol for 10 s, rinsed with ddH_2_O for 1 min, blue-colored in 0.6% ammonia water for 5 s, and rinsed with ddH_2_O.

***RT-PCR***

Total RNA was extracted from the pancreatic samples using a TRIzol kit (Promega, Madison, WI, USA). The extracted RNA was reverse-transcribed into cDNA. The RT-PCR reaction conditions were as follows: activation of UDG enzyme at 50 ℃ for 2 min, pre-denaturation at 95 ℃ for 2 min, denaturation at 95 ℃ for 15 s, annealing at 60 ℃ for 1 min, for a total of 40 cycles. In this experiment, the relative gene expression level was calculated using the 2^- Δ Δ CT^ method, where Δ Δ CT=Δ CT (target gene) - Δ CT (internal reference), and GAPDH was used as the internal reference. The primers used for RT-PCR are listed in Table S2.

***Enrichment analysis***

The KEGG pathway, gene ontology (GO) functional, and reactome functional enrichment analyses of differentially expressed genes were performed using ClusterProfiler v3.4.4. Representative dysregulated genes between the GSDMD-/- AP and WT AP groups were shown in Table S3.

***Histological scoring for acute pancreatitis***

As previously described, histopathological scores for acute pancreatitis were assessed based on edema, inflammatory cell infiltration, and necrosis[3, 4]. A comprehensive histopathology score was obtained by adding the individual scores for these features. Three separate and blinded evaluators evaluated 10 randomly selected areas in each pancreatic section.

***Immunofluorescence***

The paraffin slices were baked in the oven at 60 ℃ for 30 min, dewaxed in xylenes, 100% ethanol and 95% ethanol, successively, and then placed in 0.01M citric acid buffer for antigen repair. After sealing with 3% bovine serum albumin (BSA), the slices were incubated with the GSDMD antibody (1:200, cat. no. ab219800, Abcam, Cambridge, UK), caspase-1 antibody (1:200, cat. no. ab207802, Abcam, Cambridge, UK) and incubated at 4 ℃ overnight. The following day, the secondary antibody was added for incubation. After staining with DAPI, the tablets were sealed with anti-fluorescence quenching tablets, and images were observed under an inverted fluorescence microscope.


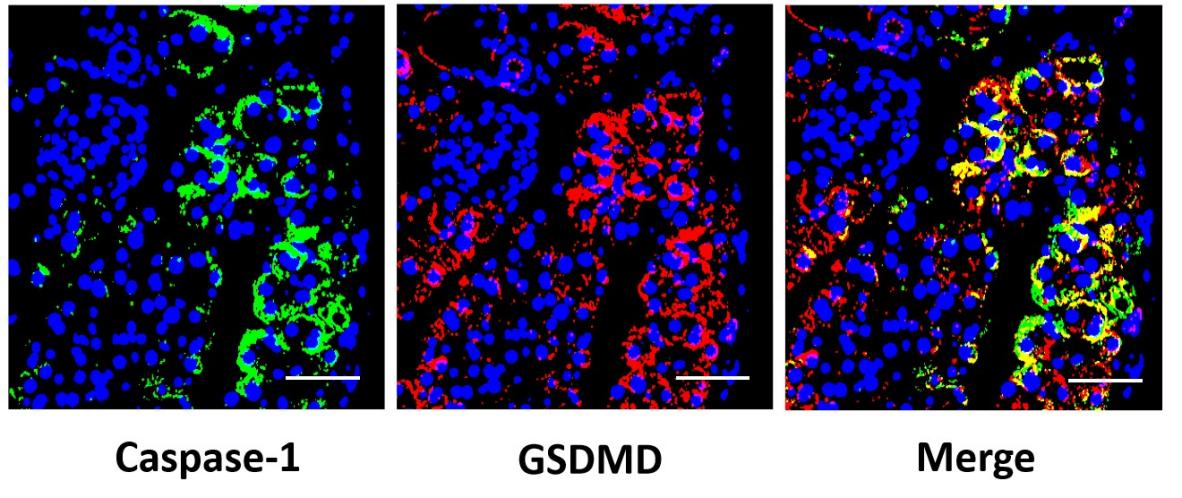


**Figure S1. GSDMD-mediated pyroptosis is activated in mice acute pancreatitis.** Representative immunofluorescence images of caspase-1 and GSDMD co-expression in pancreatic tissue of the AP group. Abbreviation: GSDMD, gasdermin D; AP: acute pancreatitis. Scale bars: 20 μm.


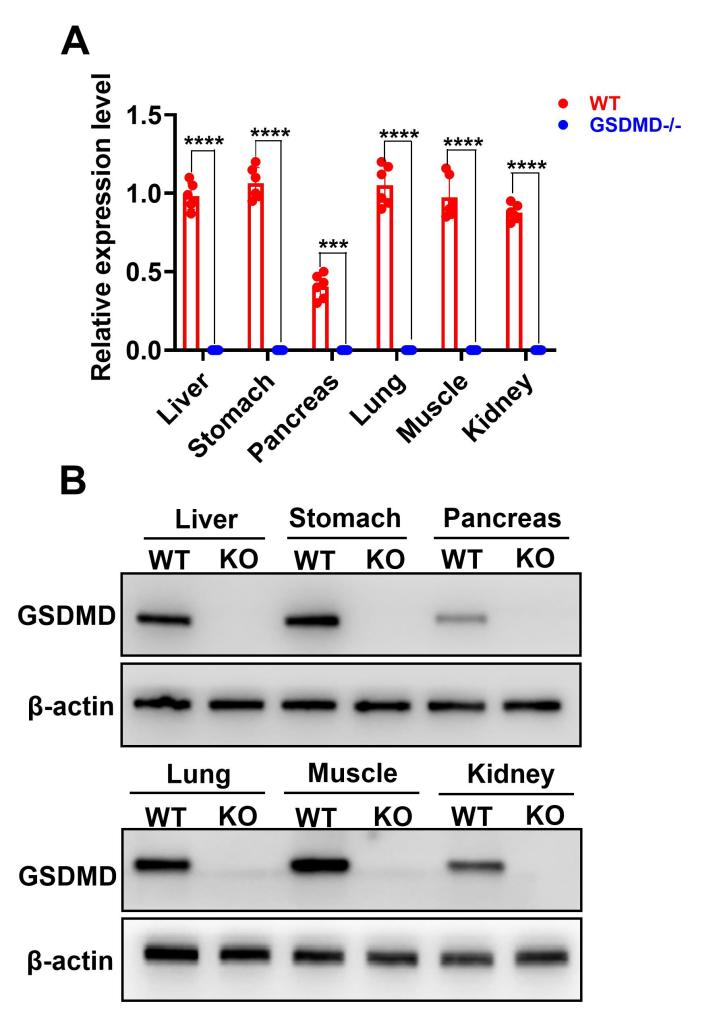


**Figure S2. (A) RT-PCR and (B) Western blotting analysis of GSDMD expression in different tissues from WT and KO mice.** Abbreviation: GSDMD, gasdermin D; WT, wild-type mice; KO, GSDMD-/- mice. *p*-values were determined by two-tailed unpaired Student's t-test (A). *****p*<0.0001, ****p*<0.001.


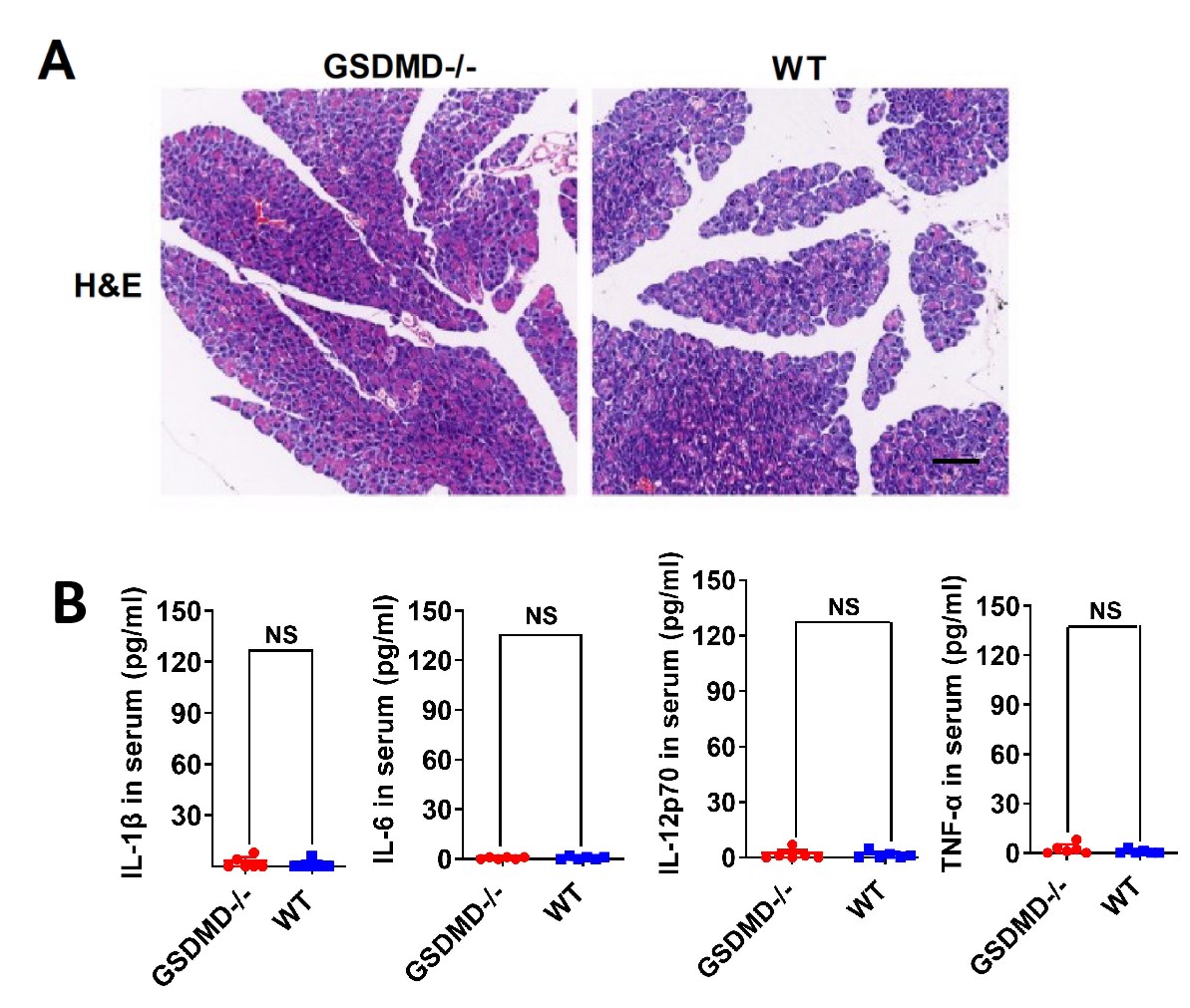


**Figure S3. There is no significant difference in pancreatic pathological morphology and inflammatory indexes between GSDMD-/- and wild-type control mice.** (A) Representative images of hematoxylin and eosin (H&E)-stained sections for GSDMD-/- and WT mice at 7 weeks of age. There was no significant difference in pancreatic histology between GSDMD-/- and WT mice. Scale bars: 50 μm. (B) Levels of serum inflammatory factors in the GSDMD-/- and WT groups. There was no significant difference in inflammatory indexes between the GSDMD-/- (n=6) and WT groups (n=6). Abbreviation: WT, wild-type; GSDMD, gasdermin D; NS, not significant. *p*-values were determined by two-tailed unpaired Student's t-test (B).


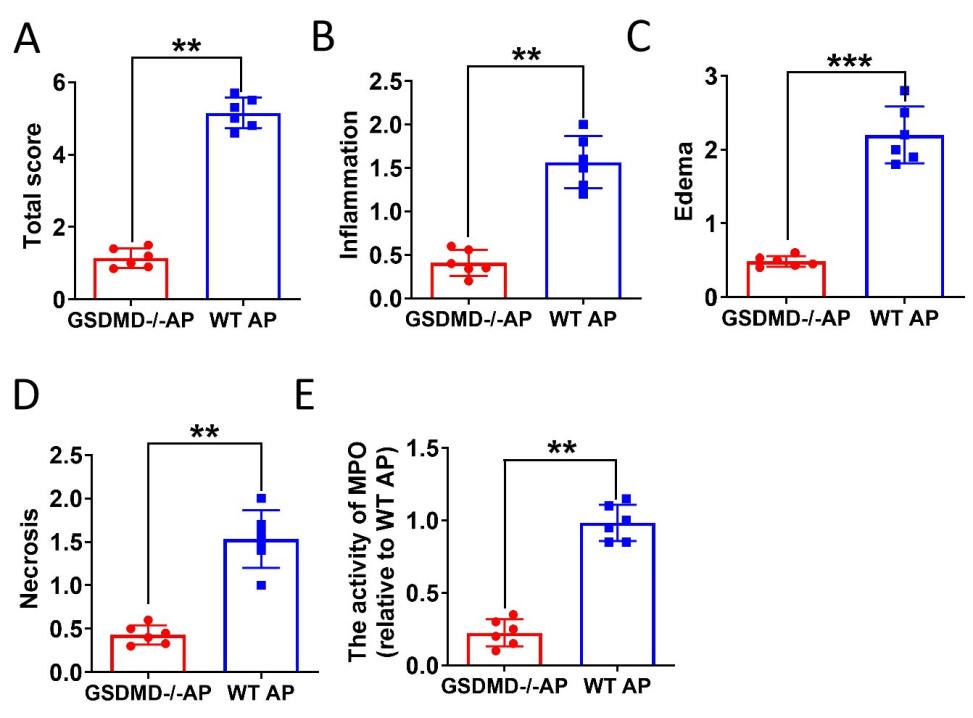


**Figure S4. GSDMD knockout reduces the pathological score of mice with pancreatitis.** (A-E) The histological evaluation of pancreatic inflammatory infiltration, edema, necrosis, the activity of myeloperoxidase (MPO) and overall sum of these features. Abbreviation: AP, acute pancreatitis; WT, wild-type; GSDMD, gasdermin D. *p*-values were determined by two-tailed unpaired Student's t-test (A-E). ****p*<0.001, ***p*<0.01.


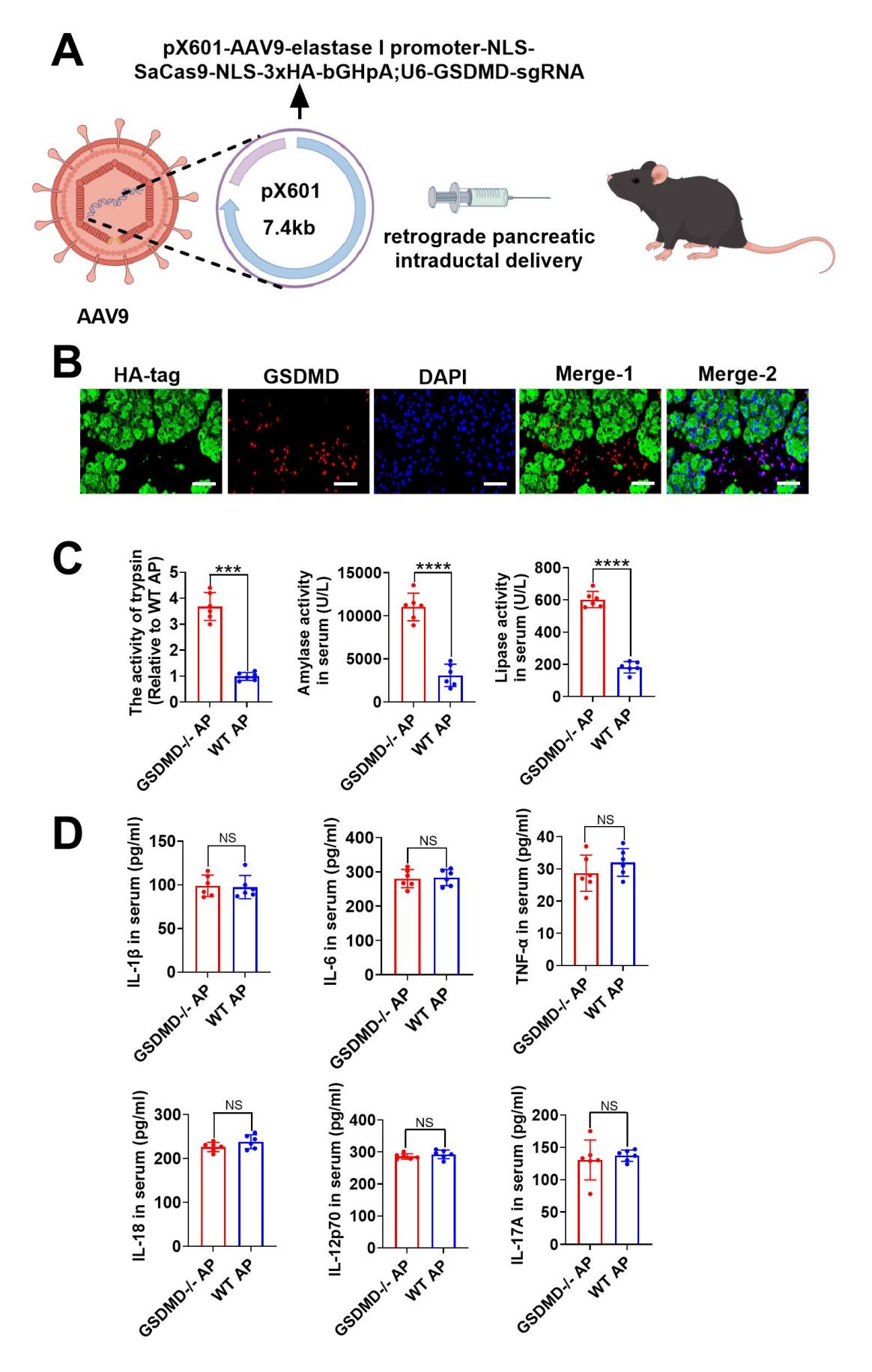


**Figure S5. GSDMD knockout in acinar cells increased the synthesis of pancreatic enzymes *in vivo*.** (A) Schematic of the HA-tagged acinar cell-specific AAV9- elastase I- GSDMD-sgRNA adeno-associated virus structure. (B) Representative immunofluorescence images of GSDMD expression in acinar cell expressing the HA-tag. Scale bars: 50 μm. (C) Changes in serum trypsin, amylase, and lipase activities in the GSDMD-/- AP (n=6) and the WT AP (n=6). (D) Levels of serum inflammatory cytokines in the GSDMD-/- AP (n=6) and WT AP groups (n=6). Abbreviation: NS, not significant; WT, wild-type; AP, acute pancreatitis. *p*-values were determined by two-tailed unpaired Student's t-test (C-D).*****p*<0.0001, ****p*<0.001.

**
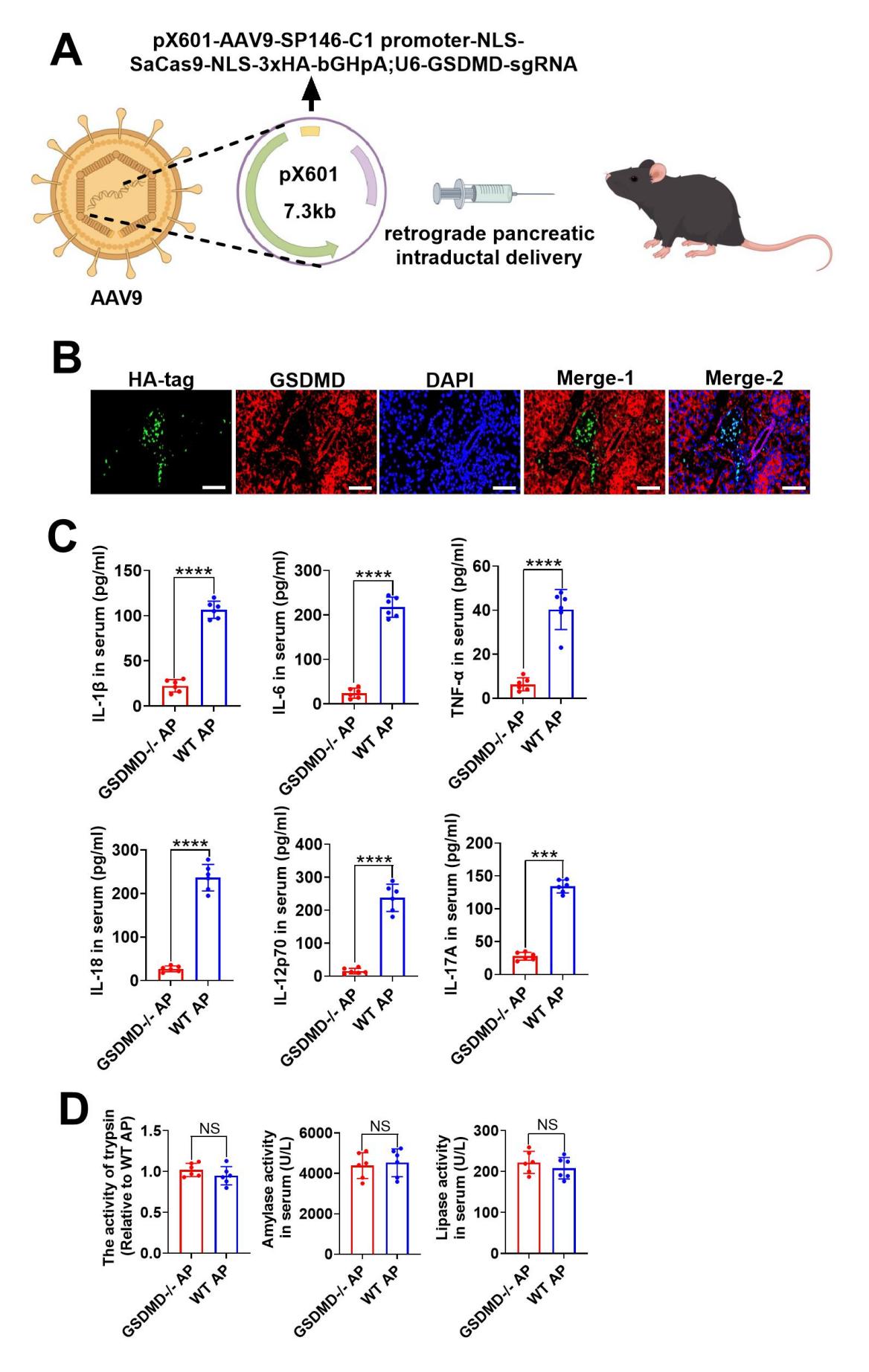
**

**Figure S6. GSDMD knockout in macrophages reduced the levels of serum inflammatory cytokines *in vivo*.** (A) Schematic of the HA-tagged macrophage-specific AAV9- SP146-C1- GSDMD-sgRNA adeno-associated virus structure. (B) Representative immunofluorescence images of GSDMD expression in macrophages expressing the HA-tag. Scale bars: 50 μm. (C) Levels of serum inflammatory cytokines in the GSDMD-/- AP (n=6) and WT AP groups (n=6). (D) Changes in serum trypsin, amylase, and lipase activities in the GSDMD-/- AP (n=6) and the WT AP (n=6). Abbreviation: NS, not significant; WT, wild-type; AP, acute pancreatitis. *p*-values were determined by two-tailed unpaired Student's t-test (C-D).*****p*<0.0001, ****p*<0.001.


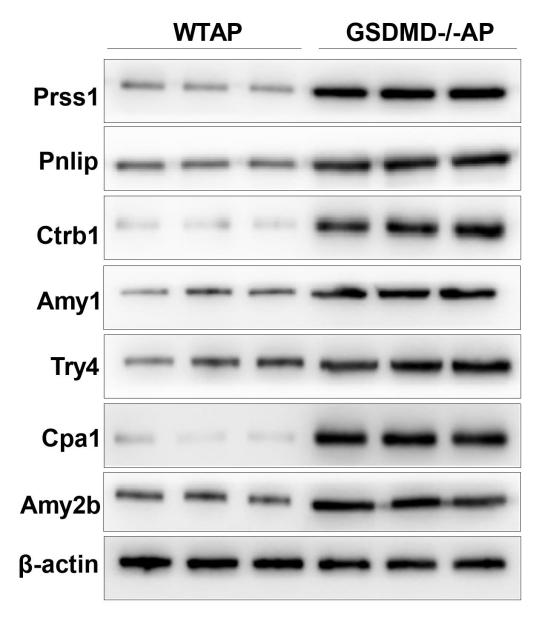


**Figure S7. Expression of pancreatic enzyme-related differential genes in GSDMD-/- AP and WT AP mice verified using Western blotting.** Abbreviation: WT, wild-type, AP, acute pancreatitis; GSDMD, gasdermin D.


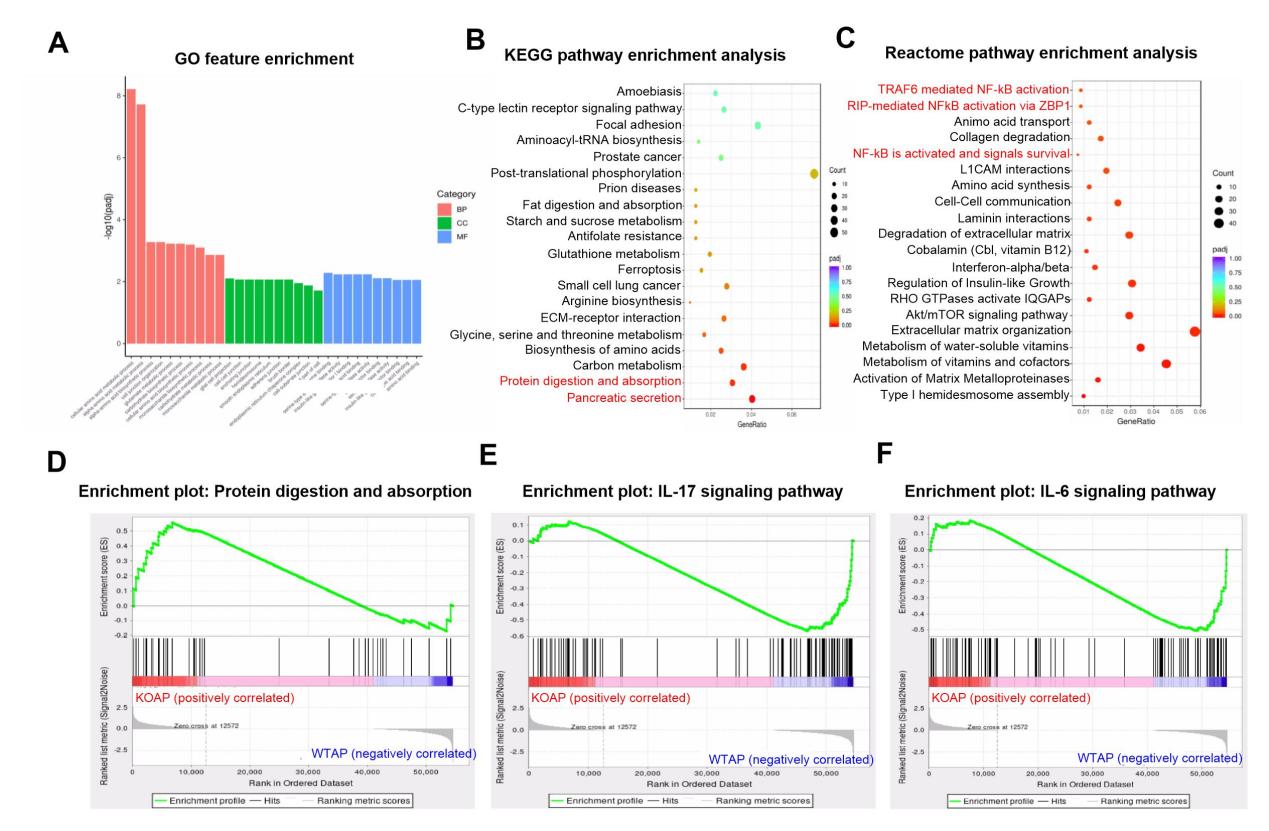


**Figure S8. The pancreatic synthesis and inflammation-related pathway were significantly enriched in the GSDMD-/- AP group.** (A) GO functional enrichment analysis showed that cellular amino acid metabolic process and alpha-amino acid metabolic process were significantly enriched. (B) The KEGG pathway enrichment analysis results showed that pancreatic secretion and the protein digestion and absorption pathway were significantly enriched. (C) The Reactome pathway enrichment analysis showed that NF-κB pathways were significantly enriched. (D-F) GSEA analysis showed that the effect of GSDMD knockout on protein digestion and absorption was enhanced, whereas the effect on IL-17 and IL-6 signaling was weakened. Abbreviation: GSDMD, gasdermin D; GO, gene ontology; KEGG, Kyoto Encyclopedia of Genes and Genomes; GSEA, gene set enrichment analysis.


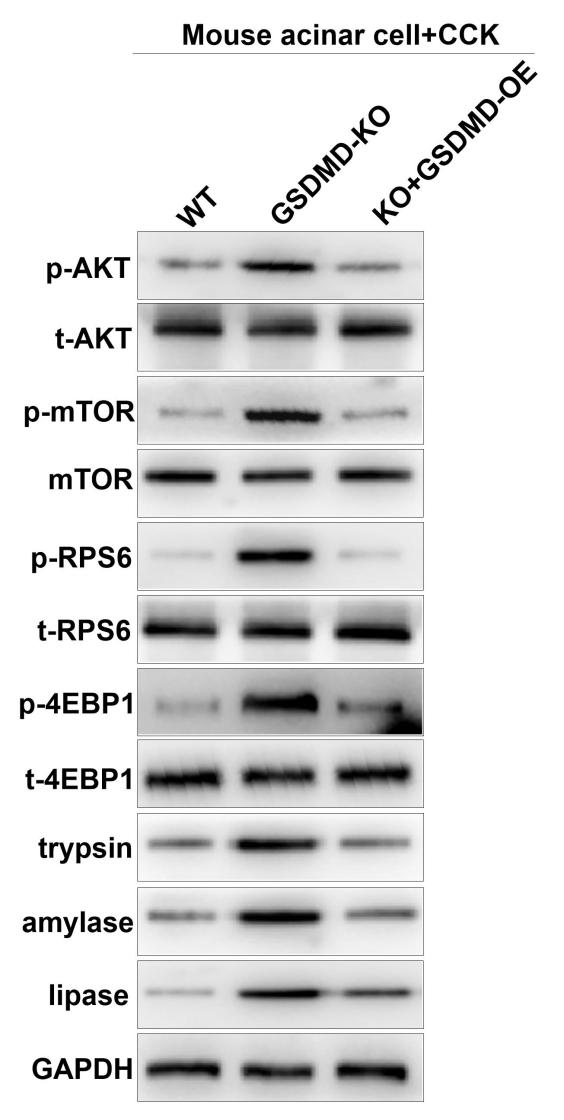


**Figure S9.** Representative Western blotting results of WT, GSDMD-KO and GSDMD-KO+GSDMD-OE cholecystokinin (CCK)-stimulated (0.001 mM) primary cultured mouse acinar cells.


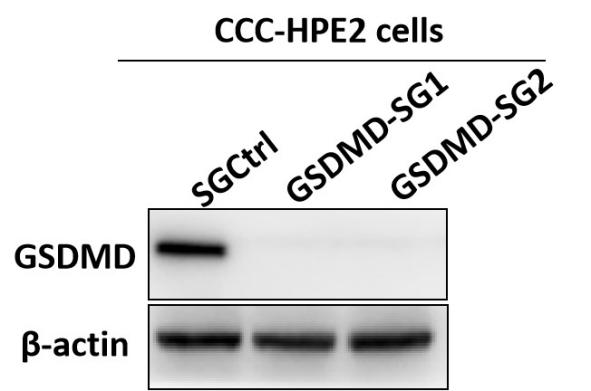


**Figure S10. Western blotting results showing the sgRNA knockout of GSDMD in CCC-HPE2 cells.** Abbreviation: GSDMD, gasdermin D.


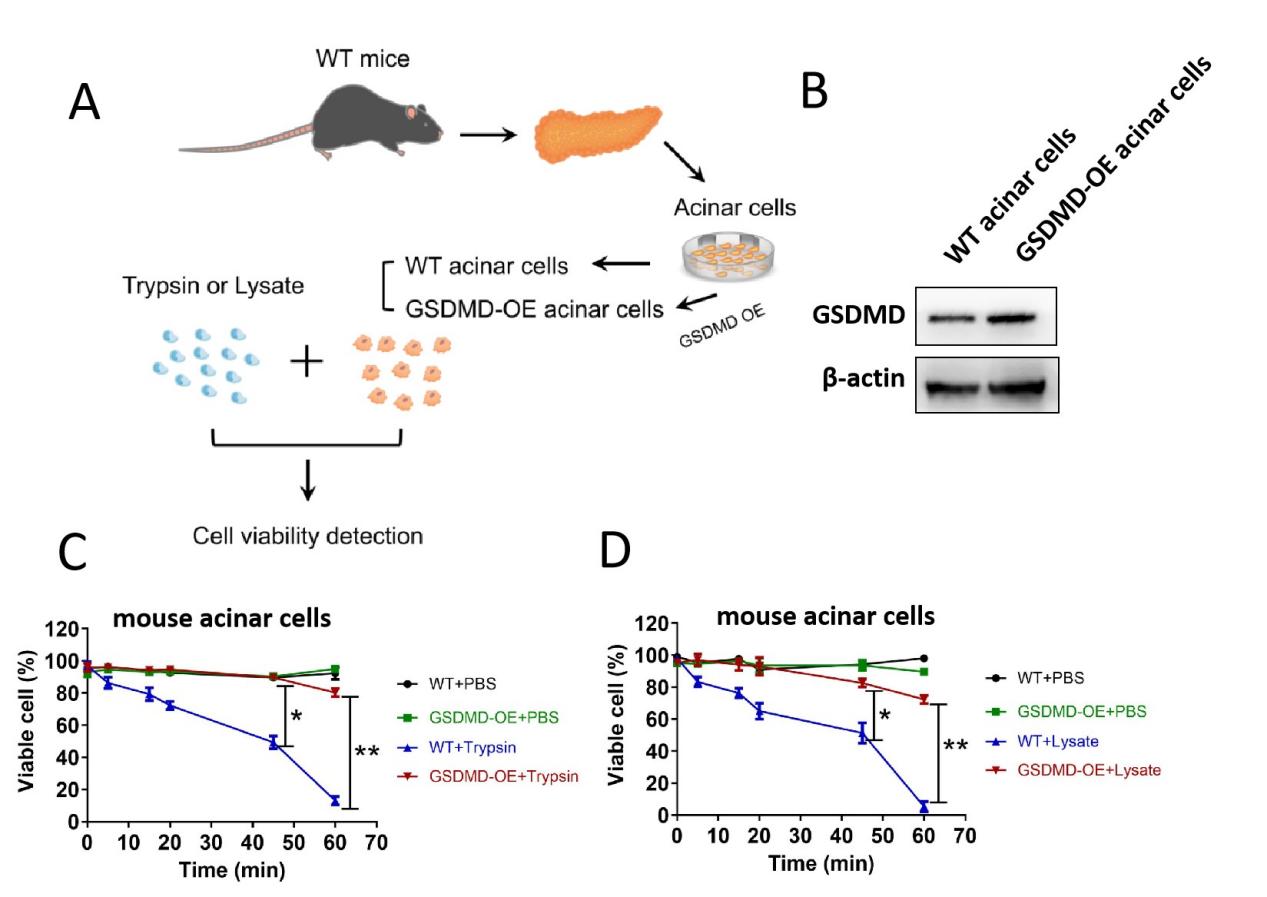


**Figure S11. GSDMD contributes to the resistance of acinar cells to trypsin.** (A) Experimental design flow diagram. (B) Immunoblotting analysis of the indicated proteins in WT and GSDMD-overexpressing (GSDMD-OE) acinar cells. (C) GSDMD-OE and WT mouse acinar cells were treated with trypsin (45 U ml^-1^). Cell viability was determined using ATP cell viability assay at 0, 5, 15, 20, 45, 60 min. (D) GSDMD-OE and WT mouse acinar cells were treated with lysate (20 μL ml^-1^) isolated from mouse pancreas. Cell viability was determined using ATP cell viability assay at 0, 5, 15, 20, 45, 60 min. Abbreviation: GSDMD, gasdermin D; WT, wild-type. *p*-values were determined by one-way ANOVA with Dunnett's multiple comparisons (C and D). **p*<0.05, ***p*<0.01.


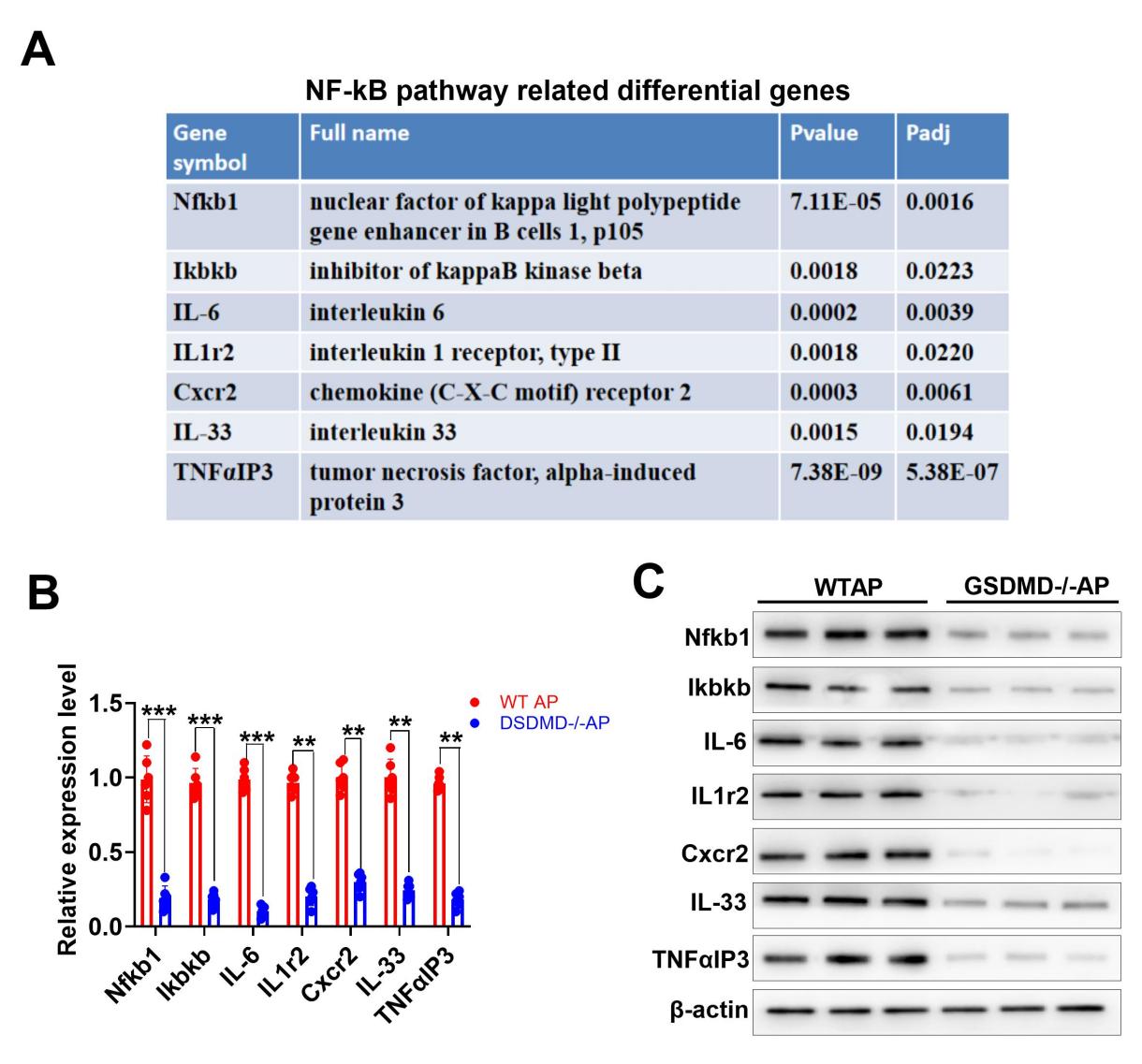


**Figure S12. GSDMD knockout weakens NF-κB signaling.** (A) NF-kB signaling-related differential genes were significantly expressed between the GSDMD-/- AP and WT AP groups. (B) The expression of NF-kB signaling-related differential genes were verified using RT-PCR. (C) The expression of NF-kB signaling-related differential genes in GSDMD-/- AP and WT AP mice were verified using Western blotting. Abbreviation: GSDMD, gasdermin D; WT, wild-type; AP, acute pancreatitis. *p*-values were determined by two-tailed unpaired Student's t-test (B). ****p*<0.001, ***p*<0.01.


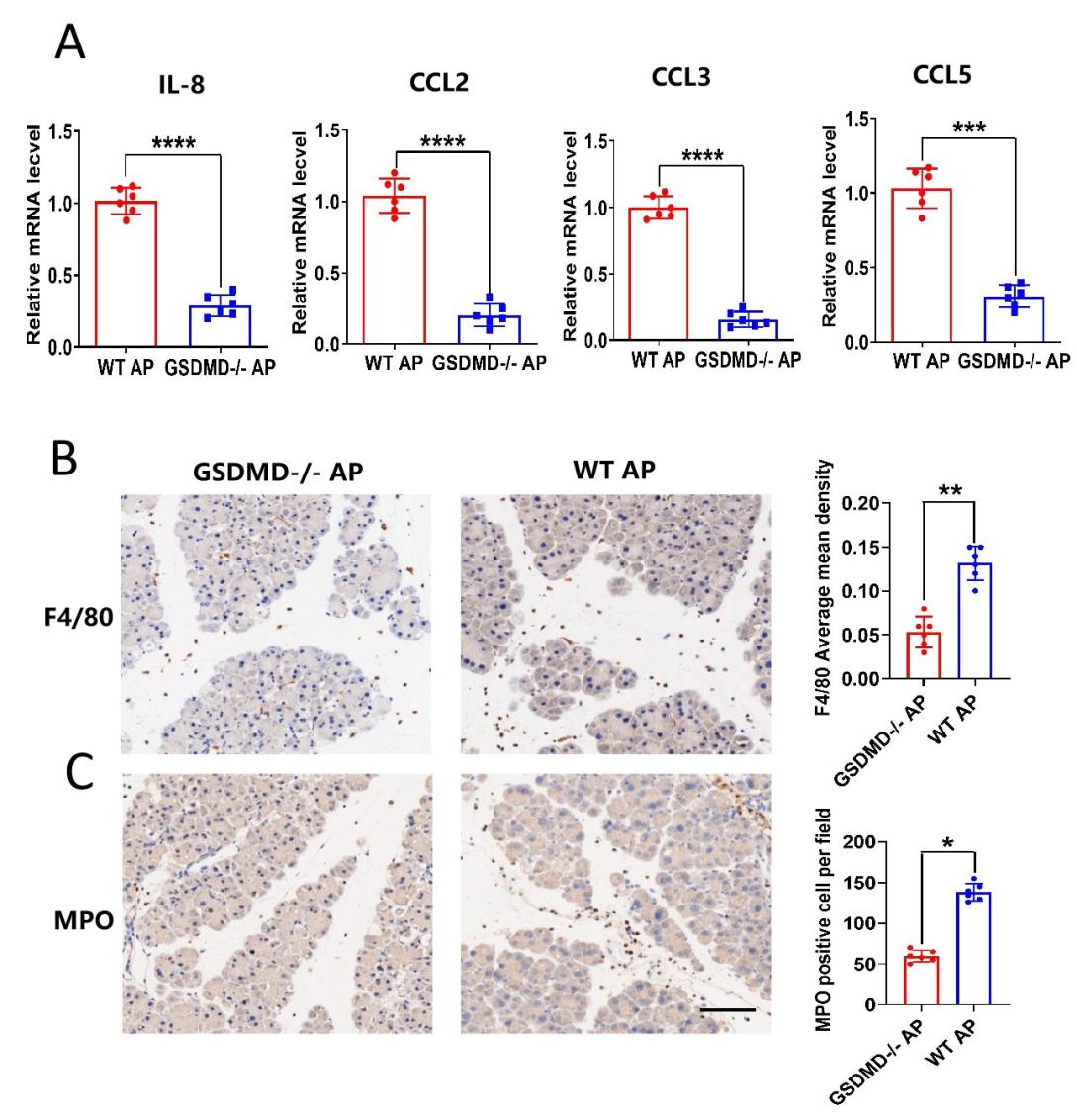


**Figure S13. GSDMD increases the infiltration of macrophages and neutrophils in acute pancreatitis.** (A) The expression levels of neutrophil chemokines (IL-8) and macrophage chemokines (e.g., CCL2, CCL3 and CCL5) in pancreatic tissues of the GSDMD-/ -AP (n=6) and WT AP groups (n=6) using RT-PCR. (B-C) Representative IHC images of F4/80 and MPO expression in pancreatic tissues of the GSDMD-/- AP (n=6) and WT AP groups (n=6). Scale bars: 50μm. Abbreviation: GSDMD, gasdermin D; MPO, myeloperoxidase; WT, wild-type; AP, acute pancreatitis. *p*-values were determined by two-tailed unpaired Student's t-test (A and B). *****p*<0.0001,****p*<0.001, ***p*<0.01, **p*<0.05.

**Table S1. Quality inspection results of sequencing data.**

| Sample | Raw reads | Clean reads | Error rate | Q20 | Q30 | GC  PCT |
| --- | --- | --- | --- | --- | --- | --- |
| GSDMD-/-  AP1 | 68317410 | 67372686 | 0.03 | 97.35 | 92.59 | 51.49 |
| GSDMD-/-  AP2 | 70022900 | 69450626 | 0.03 | 97.95 | 93.89 | 51.2 |
| GSDMD-/-  AP3 | 79549342 | 78673024 | 0.03 | 97.91 | 93.84 | 51.09 |
| WT AP1 | 72642810 | 71869226 | 0.03 | 97.93 | 93.91 | 50.88 |
| WT AP2 | 78220776 | 77390484 | 0.03 | 97.93 | 93.91 | 50.95 |
| WT AP3 | 86243204 | 84858398 | 0.03 | 97.87 | 93.76 | 51.13 |

**Table S2. Sequences of primers**

| Primer name |  | Sequences |
| --- | --- | --- |
| IL6 | forward primer: | 5’- TGGGGCTCTTCAAAAGCTCC-3’ |
|  | reverse primer: | 5’- AGGAACTATCACCGGATCTTCAA-3’ |
| IL1r2 | forward primer: | 5’- TGGGTGAAGGGTAACATACTCTGGA-3’ |
|  | reverse primer: | 5’- CCGTAGCTTGGTCAGTCCAT-3’ |
| Cxcr2 | forward primer: | 5’- ATGCCCTCTATTCTGCCAGAT-3’ |
|  | reverse primer: | 5’- GTGCTCCGGTTGTATAAGATGAC-3’ |
| Nfkb1 | forward primer: | 5’- GGAGGCATGTTCGGTAGTGG-3’ |
|  | reverse primer: | 5’- CCCTGCGTTGGATTTCGTG-3’ |
| Ikbkb | forward primer: | 5’- ACAGCCAGGAGATGGTACG -3’ |
|  | reverse primer: | 5’- CAGGGTGACTGAGTCGAGAC -3’ |
| IL33 | forward primer: | 5’- TCCAACTCCAAGATTTCCCCG-3’ |
|  | reverse primer: | 5’- CATGCAGTAGACATGGCAGAA-3’ |
| Try4 | forward primer: | 5’- TGAGCAGTTTGTCAATTCTGCC-3’ |
|  | reverse primer: | 5’- GCATGATGTCGTTGTTCAGGG-3’ |
| Amy1 | forward primer: | 5’- GCAAGTGGGATGGAGAAAAGA-3’ |
|  | reverse primer: | 5’- CACCCGTGTGAAACCATAAGG-3’ |
| Pnlip | forward primer: | 5’- CTGGGAGCAGTAGCTGGAAG-3’ |
|  | reverse primer: | 5’- AGCGGGTGTTGATCTGTGC-3’ |
| GAPDH | forward primer: | 5’- AGGTCGGTGTGAACGGATTTG-3’ |
|  | reverse primer: | 5’- TGTAGACCATGTAGTTGAGGTCA-3’ |

**Table S3. List of representative dysregulated genes between the GSDMD-/-AP and WT AP groups**

| Gene symbol | Full name | Log_2_FlodChange | Pvalue | Padj |
| --- | --- | --- | --- | --- |
| C7 | complement component 7 | -1.1804 | 8.75E-05 | 0.0019 |
| Il6 | interleukin 6 | -1.9584 | 0.0002 | 0.0038 |
| MUC1 | mucin 1 | -2.0260 | 4.93E-09 | 3.69E-07 |
| Cd209a | CD209a antigen | -1.8959 | 3.36E-06 | 0.0001 |
| Nr0b2 | nuclear receptor subfamily 0, group B, member 2 | -1.6151 | 2.34E-11 | 2.85E-09 |
| Cxcl5 | chemokine (C-X-C motif) ligand 5 | -1.5295 | 7.01E-07 | 3.12E-05 |
| Il1r2 | interleukin 1 receptor, type II | -1.4556 | 0.0018 | 0.0220 |
| Ccl22 | chemokine (C-C motif) ligand 22 | -1.3873 | 0.0032 | 0.0339 |
| Crp | C-reactive protein, pentraxin-related | -1.3007 | 3.55E-05 | 0.0009 |
| Cxcr2 | chemokine (C-X-C motif) receptor 2 | -1.2876 | 0.0003 | 0.0061 |
| Cxcl14 | chemokine (C-X-C motif) ligand 14 | -1.2171 | 2.54E-16 | 5.93E-14 |
| Tnip1 | TNFAIP3 interacting protein 1 | -1.1047 | 1.29E-17 | 3.46E-15 |
| Tnfaip3 | tumor necrosis factor, alpha-induced protein 3 | -1.0690 | 7.38E-09 | 5.38E-07 |
| Tnfrsf18 | tumor necrosis factor receptor superfamily,  member 18 | -1.0356 | 0.0010 | 0.0153 |
| Nfkb2 | nuclear factor of kappa light polypeptide gene  enhancer in B cells 2, p49/p100 | -0.8445 | 1.10E-12 | 1.66E-10 |
| Casp7 | caspase 7 | -0.8355 | 6.15E-07 | 2.83E-05 |
| Il22ra1 | interleukin 22 receptor, alpha 1 | -0.7803 | 3.14E-11 | 3.67E-09 |
| Smad7 | SMAD family member 7 | -0.6494 | 0.0024 | 0.0280 |
| Il33 | interleukin 33 | -0.6032 | 0.0015 | 0.0194 |
| Hmga1 | high mobility group AT-hook 1 | -0.5960 | 8.66E-11 | 9.18E-09 |
| Ccl11 | chemokine (C-C motif) ligand 11 | -0.5894 | 0.0005 | 0.0076 |
| Ccl24 | chemokine (C-C motif) ligand 24 | -0.5862 | 0.0027 | 0.0304 |
| Hmga1b | high mobility group AT-hook 1B | -0.5795 | 3.09E-06 | 0.0001 |
| Cxcl16 | chemokine (C-X-C motif) ligand 16 | -0.4943 | 0.0007 | 0.0106 |
| Nfkb1 | nuclear factor of kappa light polypeptide gene  enhancer in B cells 1, p105 | -0.4527 | 7.11E-05 | 0.0016 |
| Il6ra | interleukin 6 receptor, alpha | -0.4044 | 0.0044 | 0.0429 |
| Ikbkb | inhibitor of kappaB kinase beta | -0.3925 | 0.0018 | 0.0223 |
| Litaf | LPS-induced TN factor | -0.2890 | 0.0005 | 0.0076 |
| Stat5a | signal transducer and activator of transcription 5A | -0.4324 | 0.0047 | 0.0453 |
| Cxcl13 | chemokine (C-X-C motif) ligand 13 | 2.8813 | 0.0017 | 0.0217 |
| Try5 | trypsin 5 | 0.6954 | 3.39E-09 | 2.62E-07 |
| Try4 | trypsin 4 | 1.6501 | 1.67E-54 | 5.95E-51 |
| Try10 | trypsin 10 | 0.3249 | 0.0006 | 0.0089 |
| Tlr9 | toll-like receptor 9 | 1.4242 | 0.0021 | 0.0257 |
| Prss3 | protease, serine 3 | 2.7924 | 1.45E-115 | 1.03E-111 |
| Cd22 | CD22 antigen | 2.4156 | 0.0011 | 0.0158 |
| Ptf1a | pancreas specific transcription factor, 1a | 1.7391 | 2.53E-29 | 2.40E-26 |
| Prss1 | protease, serine 1 (trypsin 1) | 1.5218 | 0.0012 | 0.0160 |
| Amy1 | amylase 1, salivary | 0.3910 | 0.0014 | 0.0185 |
| Amy2a3 | amylase 2a3 | 0.4677 | 0.0003 | 0.0049 |
| Amy2a5 | amylase 2a5 | 0.4910 | 3.12E-07 | 1.56E-05 |
| Amy2b | amylase 2b | 0.3291 | 2.28E-05 | 0.0006 |
| Pnlip | pancreatic lipase | 0.3461 | 0.0002 | 0.0046 |
| Ctrb1 | chymotrypsinogen B1 | 0.5693 | 1.35E-08 | 9.34E-07 |
| Cpa1 | carboxypeptidase A1, pancreatic | 0.6448 | 1.84E-17 | 4.84E-15 |

**References**

[1] W. Huang, M. C. Cane, R. Mukherjee, P. Szatmary, X. Zhang, V. Elliott, Y. Ouyang, M. Chvanov, D. Latawiec, L. Wen, D. M. Booth, A. C. Haynes, O. H. Petersen, A. V. Tepikin, D. N. Criddle, R. Sutton, *Gut* **2017**, *66* (2), 301, <https://doi.org/10.1136/gutjnl-2015-309363>.

[2] Y. Chen, X. Li, R. Lu, Y. Lv, Y. Wu, J. Ye, J. Zhao, L. Li, Q. Huang, W. Meng, F. Long, W. Huang, Q. Xia, J. Yu, C. Fan, X. Mo, *MedComm (2020)* **2024**, *5* (11), e686, <https://doi.org/10.1002/mco2.686>.

[3] X. Zhou, C. Xue, *Pancreas* **2009**, *38* (7), 752, <https://doi.org/10.1097/MPA.0b013e3181a86b74>.

[4] W. Du, G. Liu, N. Shi, D. Tang, P. E. Ferdek, M. A. Jakubowska, S. Liu, X. Zhu, J. Zhang, L. Yao, X. Sang, S. Zou, T. Liu, R. Mukherjee, D. N. Criddle, X. Zheng, Q. Xia, P. O. Berggren, W. Huang, R. Sutton, Y. Tian, W. Huang, X. Fu, *Mol Ther* **2022**, *30* (4), 1754, <https://doi.org/10.1016/j.ymthe.2022.01.033>.
